# Determination of chemical constituent yields in e-cigarette aerosol using partial and whole pod collections, a comparative analysis

**DOI:** 10.3389/fchem.2023.1223967

**Published:** 2023-09-07

**Authors:** J. Brian Jameson, Jiaming Wang, Patrick C. Bailey, Michael J. Oldham, Cameron R. Smith, Lena N. Jeong, David K. Cook, Austin L. Bates, Sifat Ullah, Alexander S. C. Pennington, I. Gene Gillman

**Affiliations:** Juul Labs, Inc., Washington, DC, United States

**Keywords:** ENDS, nicotine, tobacco, carbonyls, formaldehyde, metals, nickel, glycidol

## Abstract

Literature reports the chemical constituent yields of electronic nicotine delivery systems (ENDS) aerosol collected using a range of aerosol collection strategies. The number of puffs to deplete an ENDS product varies widely, but collections often consist of data from the first 50–100 puffs. However, it is not clear whether these discrete puff blocks are representative of constituent yields over the life of a pod. We aimed to assess the effect of differing aerosol collection strategies on reported yields for select chemical constituents in the aerosol of closed pod-based ENDS products. Constituents analyzed were chosen to reflect important classes of compounds from the Final Premarket Tobacco Product Application Guidance. Yields were normalized to total device mass loss (DML). Collection strategies that consisted of partial pod collection were valid for determining yields of constituents whose DML normalized yields were consistent for the duration of pod life. These included primary aerosol constituents, such as propylene glycol, glycerol, and nicotine, and whole pod yields could be determined from initial puff blocks. However, changes were observed in the yields of some metals, some carbonyl compounds, and glycidol over pod life in a chemical constituent and product dependent manner. These results suggest that collection strategies consisting of initial puff block collections require validation per chemical constituent/product and are not appropriate for chemical constituents with variable yields over pod life. Whole pod collection increased sensitivity and accuracy in determining metal, carbonyl, and glycidol yields compared to puff block-based collection methodologies for all products tested.

## 1 Introduction

The United States Food and Drug Administration (FDA) Premarket Tobacco Product Application (PMTA) Guidance for Electronic Nicotine Delivery System/s (ENDS) recommends that ENDS manufacturers measure a range of chemical constituents in aerosol to support PMTAs for new ENDS products (United States Food & Drug A dministration, 2019). While this guidance specifies a number of chemical constituents that should be included for analysis, appropriate methodologies for ENDS aerosol collection are not specified. In the case of combustible cigarettes (CC), research has been conducted to determine appropriate experimental conditions for the collection of mainstream smoke for chemical analysis. Multiple studies have shown that puffing topography affects mainstream smoke chemical constituent yields (Counts et al., 2005; Klus et al., 2016). Similarly, a number of studies that have used specialized techniques to measure the chemical constituent emissions in mainstream CC smoke on a puff-by-puff basis have reported changes from the first puff to the last puff (Bush et al., 1972; Parrish et al., 2001; Thomas and Koller, 2001; Wagner et al., 2005; Adam et al., 2007; Crawford et al., 2007; Thweatt et al., 2007; Ceschini and Lafaye, 2014). To facilitate product comparison, standard puffing regimens and aerosol collection techniques were created for CC (International Organization for Standardization, 1977; TobLabNet, 2012; Rodgman and Perfetti, 2013; Pazo et al., 2016; Bitzer et al., 2018; Engineering, 2018; Crosswhite et al., 2021). Based upon these standardized techniques, reporting whole cigarette yields is the standard for ISO (International Organization for Standardization, 2021), FTC (NCI, 1996), Health Canada (Canada, 2018), and WHO (TobLabNet, 2014) methods, and the analysis of CC smoke is most often reported on a whole cigarette basis (Vu et al., 2015; Pazo et al., 2016; Edwards et al., 2017). Collection and reporting standards for CC have facilitated thorough characterization of the chemical composition of CC smoke (Calafat et al., 2004; Rodgman and Perfetti, 2013; Li and Hecht, 2022; Soleimani et al., 2022).

ENDS products generate a nicotine-containing aerosol below combustion temperatures and generally comprise a battery, a heating element and a reservoir for storing nicotine-containing liquid (Bitzer et al., 2018). ENDS products that use replaceable non-refillable reservoirs are termed closed pod-based ENDS. Because of the dramatic differences in design between CC and ENDS, ENDS specific methods for puffing, aerosol collection, and total yield determination have been developed (Cheng, 2014). For example, researchers have reported ENDS aerosol collection methodologies ranging from 15 to 150 puffs and reported the levels of ENDS aerosol chemical constituents in multiple formats including per puff (Flora et al., 2017; El-Hellani et al., 2018), per mg nicotine (Talih et al., 2019; Chen et al., 2021), and per collection (Goniewicz et al., 2014; Tayyarah and Long, 2014; Margham et al., 2016). Normalization of chemical constituent measurements to aerosol mass (Li et al., 2021) or device mass loss (DML) (McAdam et al., 2021) has been used previously to account for a range of product yields. While each of these reporting formats have strengths and limitations, overall experimental design characteristics and their impact on the reported constituent yields are important considerations (Belushkin et al., 2018; Soulet and Sussman, 2022a; Soulet and Sussman, 2022b).

To design collection strategies that accurately estimate total chemical constituent yields from ENDS, it is necessary to understand whether changes in the yield profile of chemical constituents occur over the life of a product. Gupta et al. showed aerosol collected mass per puff from closed pod-based ENDS products can decrease by over 50% from first 50-puffs to last 50-puffs (Gupta et al., 2021). Belushkin et al. (2020) and colleagues tested a broad range of commercially available ENDS products and reported that formaldehyde levels can increase significantly on a per puff basis over the life of a pod in some closed system ENDS. Guthery reported the yields of a number of carbonyl chemical constituents may increase over pod life (measured over 200 puffs in 40 puff blocks), using an in-house designed non-commercial device they found that formaldehyde could increase by as much as 4,500% from the first to last puff block, acetaldehyde increased by as much as 15,900%, acrolein by as much as 9,100%, and propionaldehyde by as much as 11,500% (Guthery, 2016). However, this study was conducted using a custom device and not a commercial product, these results cannot be replicated by other researchers. These published studies indicate that aerosol collection strategies that only collect the beginning puffs from closed pod-based ENDS may not accurately reflect total aerosol constituent yields over the life of the pod. Relatedly, even when a large number of puffs are collected, such as those of Goniewicz et al. (2014) (150 puffs) and Tayyarah and Long, 2014 (99 puffs) (Goniewicz et al., 2014), aerosol generated from the remaining e-liquid in the cartridge may produce significantly different levels of chemical constituents compared to levels resulting from the initial puffs (Belushkin et al., 2020), suggesting inconsistent collection strategies may lead to inconsistency in reported yields for the same chemical constituent and product.

The present work seeks to determine in closed pod-based ENDS to what extent the total yields (i.e., the yields of a chemical constituent over the life of a pod) of primary aerosol constituents (glycerol, propylene glycol, menthol, nicotine, and water), selected metals, selected carbonyls, and glycidol are represented by different aerosol collection strategies (i.e., beginning puffs, multiple collections over pod life, or whole pod collection). One tobacco and one mint/menthol pod variant from four market leading closed pod-based ENDS manufacturers (eight total products) was used to assess the yields of the aerosol chemical constituents listed above while varying the number of puffs collected and selection of puffs blocks over the life of a pod. Through these studies we determined the impact of the aerosol collection strategy on reported total chemical constituent yields.

## 2 Materials and methods

Closed pod-based ENDS products from four market leading ENDS manufacturers (JUUL, MyBlu, NJoy Ace, and Vuse Alto) along with one tobacco and one mint or menthol flavor pod variant from each manufacturer were used in the current study. JUUL, NJoy, and Vuse products had a labeled nicotine strength of 5% and MyBlu was 2.4% nicotine strength. All were commercial products sourced from the US or Canadian markets and were available for retail purchase as of the initiation of this study in 2020. Juul Labs, Inc. suspended commercial sales of Mint flavored pods in the US market in November 2019. Aerosol collection and constituent analyses were performed by Enthalpy Analytical LLC (800 Capitola Drive, Suite 1, Durham, NC, 27713). Enthalpy Analytical LLC was accredited to the International Organization for Standardization (ISO) 17025 standard at the time of this study. All methods for chemical constituent analytical measurements were validated for the analysis of ENDS aerosol according to ICH guidance Q2 (R1) [International Council on Harmonisation. Harmonised Tripartite Guideline: Validation of Analytical Procedures, Text and Methodology; ICH: Geneva, Switzerland, 2005]. Method validations included an assessment of accuracy, precision, repeatability, intermediate precision, specificity, detection limit, quantitation limit, linearity, and recovery from the collection systems. All method validations were reviewed by an independent accreditation body as part of the ISO 17025 accreditation process. Additional method specifics (i.e., extract concentrations, internal standard concentrations, etc.) can be addressed by Enthalpy Analytical LLC upon request.

### 2.1 Pod end of life (EOL) determination

To determine the end of pod life and to minimize the contribution of dry puffs to the analytical results (Farsalinos et al., 2015), an EOL study was performed to determine the number of machine puffs needed to fully deplete each tested pod (Belushkin et al., 2020; Crosswhite et al., 2021; Crosswhite et al., 2022). EOL was determined for all pods with five replicates each. The puffing regime consisted of a 55 mL puff volume, a 3 s puff duration, and 30 s between puffs (ISO 20768:2018 standard). Aerosol was collected in 50-puff blocks. Before and after each puff block, the devices were removed from the smoking machine and weighed. Pre-puffing and post-puffing device weights were used to determine the DML for each puff block. To ensure battery depletion did not affect device performance near EOL, device batteries were replaced every puff block with fully charged batteries. All puffing was performed using a Cerulean SM450 E linear smoking machine. For EOL determination, puff blocks were collected until the DML of two consecutive puff blocks were <10 mg (typical yields shown in Supplementary Tables S1, S2). The cumulative DML at EOL was defined as the total DML prior to puff blocks yielding <10 mg. The cumulative DML was used to calculate the puff number at which 85% of total mass loss was achieved and puff one to 85% EOL was defined as whole pod. 85% of cumulative DML was selected for whole pod because one standard deviation in EOL DML is less than 15% in most cases (Supplementary Tables S1, S2), suggesting that dry puffing due to liquid exhaustion is less likely to occur below 85% of total aerosol yield.

### 2.2 Collection of ENDS aerosol

Four separate aerosol collections were performed for each pod and chemical constituent quantitation method. These separate collections consisted of a whole pod collection incorporating all aerosol from puff 1 to 85% EOL, and the beginning (puffs 1–50), middle (50 puffs ending at 50% total DML), and end (50 puffs ending at 85% total DML) puff blocks. Due to varying trapping protocols for the chemical constituent measurements, separate aerosol samples were collected for quantitation of primary constituents, selected metals, selected carbonyls, and glycidol. Five aerosol sample replicates were collected for each quantitative measurement. Thus, 80 individual aerosol sample collections were performed with each product (eight total device/flavor combinations).

### 2.3 Quantitation of primary constituents in ENDS aerosol

Aerosol was collected using glass fiber filter pads and extracted in a solution of 2-propanol containing 1,4-butanediol and quinolone (internal standards) according to Enthalpy Analytical LLC method AM-201/AM-224, similar to CORESTA method 84 (CORESTA, CRM No 84, 2021). Extracts were analyzed for glycerol, propylene glycol, menthol, nicotine, and water via a gas chromatograph equipped with a flame injection detector and thermal conductivity detector using an Agilent DB-ALC1 30 m × 0.32 mm × 1.8 µm capillary column.

### 2.4 Quantitation of selected metals in ENDS aerosol

Aerosol extract samples were collected using a Halder Process Solutions HPS-EP5 electrostatic precipitator (EP) system (Halder Process Solutions, Moseley,VA, United States), comprised of five individual tungsten electrode tips connected to a single power supply. The EP system was designed to connect directly to a linear 20-port smoking machine, while encased within a custom laminar flow hood (Enthalpy Analytical, Henrico,VA, United States). Aerosol samples were collected directly into acid-washed quartz EP tubes. Each tube was then rinsed with semi-conductor grade methanol to extract the collected aerosol. Aerosol extract samples were digested with a concentrated nitric acid solution and analyzed for arsenic, beryllium, cadmium, chromium, cobalt, copper, iron, lead, nickel, selenium, silver, tin, and zinc via inductively coupled plasma mass spectrometry according to Enthalpy Analytical LLC method AM-249, similar to CORESTA method 98 (CORESTA, CRM No 98, 2022).

### 2.5 Quantitation of selected carbonyls in ENDS aerosol

Aerosol extract samples were collected by passing aerosol through a glass fiber filter pad and a single impinger containing a 1:1 solution of acetonitrile, isopropyl alcohol, and internal standards kept at −35°C by submersion in a water/methanol bath. Following collection, the filter pad was extracted in the impinger solution and the mixture was derivatized with 2,4-dinitrophenylhydrazine. Samples were analyzed for acetaldehyde, acetyl propionyl, acrolein, n-butyraldehyde, crotonaldehyde, diacetyl, and formaldehyde according to Enthalpy Analytical LLC method AM-244, similar to CORESTA method 96 (CORESTA, CRM No 96, 2021), via an ultra-performance liquid chromatograph equipped with a tandem mass spectrometry detector in SIR mode using a Waters Acquity BEH C18, 2.1 mm × 50 mm column, with 1.7 μm pore size.

### 2.6 Quantitation of glycidol in ENDS aerosol

Aerosol extract samples were collected by passing aerosol through an impinger containing a trapping solution with internal standard, hydrochloric acid, and p-toluenesulfonyl chloride. Glycidol was extracted from the trapping solution with hexane and analyzed via a gas chromatograph equipped with a mass spectrometry detector using a 5 m section of Restek Rxi-17Sil MS 0.25 mm o.d. × 0.25 µm column followed by a Restek Rxi-5Sil MS 30 m × 0.25 mm × 0.5 µm column according to Enthalpy Analytical LLC method ENT203. At the time of initial sample collection, a suitable method for glycidol analysis of samples with >1 g of total aerosol was not available. Whole pod glycidol data for products with >1 g total DML (MyBlu, NJoy Ace, and Vuse Alto) was collected separately with an additional batch of samples. The numbers of puffs required to achieve 85% EOL for these samples were different from those used for other collections (Supplementary Table S2).

### 2.7 Data analysis

Chemical constituent measurements were normalized on a per gram DML basis to assess differences in yields between beginning puffs, multiple collections over pod life, or whole pod collection strategies for determination of chemical constituent yields. Since a consistent EOL approach was used for all products (excepting whole pod glycidol collections), constituent yields were also reported on a per puff basis. Any aerosol sample having <10 mg (0.2 mg/puff for a 50-puff collection) of total DML was excluded from chemical constituent yield calculations to minimize the contribution of dry puffing or devices that failed to puff. For aerosol samples above 10 mg of total DML, when replicate chemical constituent measurements were below the limit of quantitation (BLOQ), the arithmetic mean of the limit of detection (LOD) and limit of quantitation (LOQ) was used. For chemical constituent measurements below the limit of detection (BLOD), the LOD was divided by two. When chemical constituent measurements consisted of replicate measurements both above LOQ and BLOQ (e.g., two replicates above LOQ and three BLOQ), the arithmetic mean was computed and reported only if it exceeded the LOQ. When shown in figures, BLOD and BLOQ values are represented by the appropriate limit without error bars. In order to compare multiple collections over pod life to a whole pod collection, measurements from the beginning, middle, and end puff blocks were summed and divided by either the summed DML (Eq. 1) or puff counts (Eq. 2) for each replicate. The total chemical constituent yields based on summed beginning, middle and end puff blocks were termed estimated whole pod (EWP). In order to compare a single collection comprising a fixed number of puffs to whole pod collection, measurements from the beginning puff block in per DML or per puff format were used.

$$EWP\;per\;DML=\frac{\left(Beginning\;Yield+Middle\;Yield+End\;Yield\right)}{\left(Beginning\;DML+Middle\;DML+End\;DML\right)}$$ (1)

$$EWP\;per\;Puff=\frac{\left(Beginning\;Yield+Middle\;Yield+End\;Yield\right)}{\left(Beginning\;Puff\;Count+Middle\;Puff\;Count+End\;Puff\;Count\right)}$$ (2)

## 3 Results

### 3.1 EOL and DML determination

**FIGURE 1:**
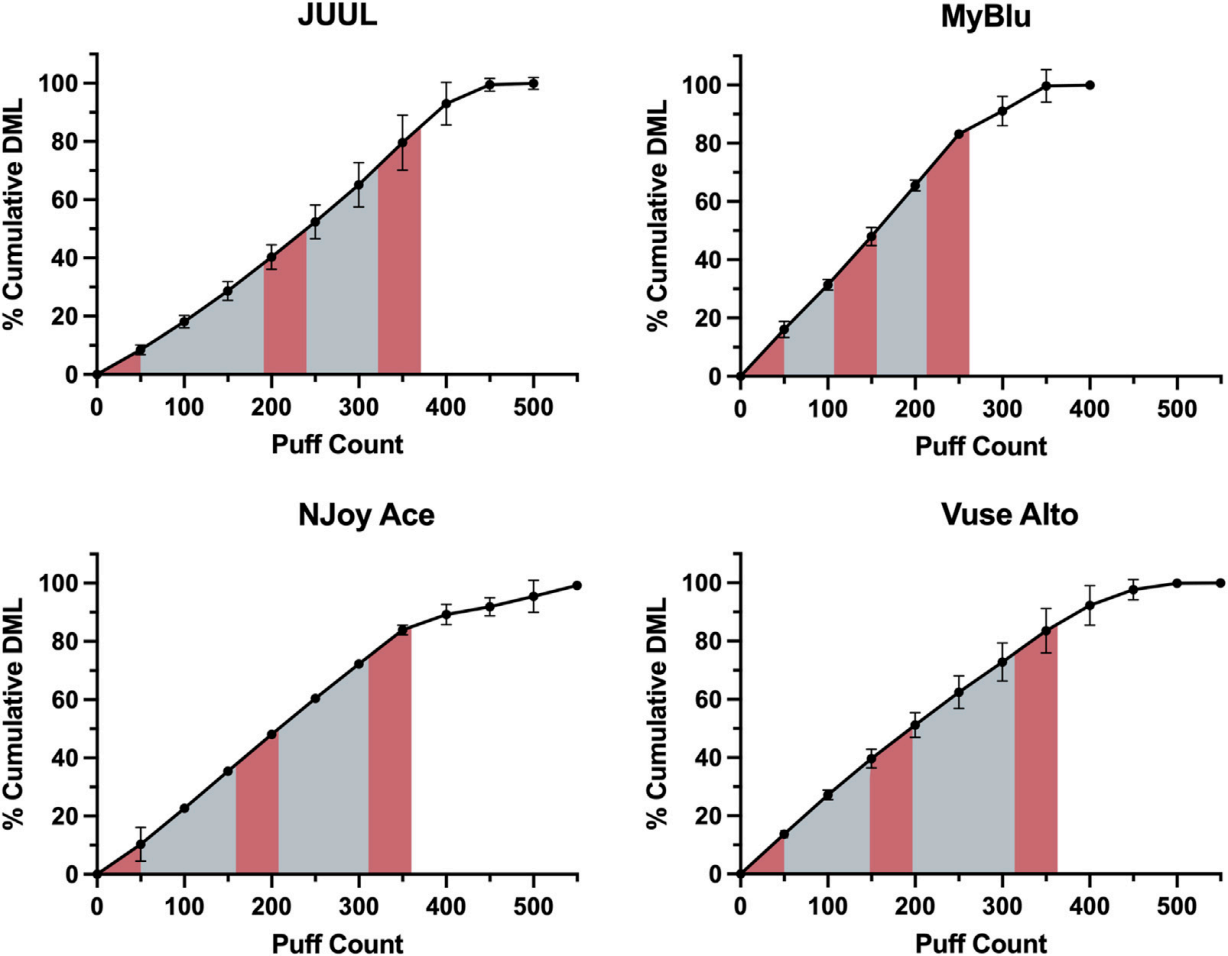
EOL determination for Tobacco flavor from each manufacturer using cumulative DML determined in 50-puff blocks. Beginning, middle, and end puff blocks are represented by red shading, whole pod collection is represented by the full shaded area.

EOL and total DML were determined for each of the eight products tested (Supplementary Tables S1, S2). An average of 350 puffs were needed to reach 85% EOL. While whole pod collection aimed to collect 85% of total DML for all products, collection of a single block of 50 puffs at the beginning of pod life represented from 7% to 18% of total DML depending on product. Collection of 150 puffs in beginning, middle and end 50-puff blocks represented from 28% to 53% of total DML. Due to the unique DML per puff yield of each product over pod life, collections based on a single or multiple fixed puff blocks represented differing proportions of total aerosol for each product (Figure 1).

### 3.2 Impact of DML on method sensitivity

Whole pod collections encompass greater aerosol mass than beginning puffs or multiple collections over pod life (Supplementary Tables S1, S2), which can impact method sensitivity. Mean LOD and LOQ for representative chemical constituents from each analytical method were determined in per gram DML format to compare method sensitivity using a single 50-puff block and whole pod collection (Table 1). Due to higher collected mass in whole pod collections, method LODs for low concentration analytes including metals, carbonyls, and glycidol were improved using whole pod collection compared to measurements comprising a single 50-puff block collection. Sensitivity is improved by higher collected mass independent of data reporting format (i.e., per puff or per DML) but detection limit improvements are only apparent when limits are normalized to aerosol mass. Method LODs for high concentration analytes (primary constituents) were not improved by whole pod collection because dilution of aerosol extracts was required to be within the linear range of the instrument method. Comparable per DML detection limits were achievable by either whole pod or 50-puff block collections for primary constituents. Instrumental detection limits per collection for all chemical constituent measurements can be found in Supplementary Table S3.

**TABLE 1:** Mean method detection limits for selected chemical constituents^a^.

		LOD		LOQ	
Chemical constituent	Units	50-Puff block	Whole pod	50-Puff block	Whole pod
Nicotine	mg/g DML	0.194	0.58	1.02	1.7
Nickel	ng/g DML	45.7	5.8	91.3	12
Formaldehyde	µg/g DML	2.54	0.7	11.4	3.1
Glycidol	µg/g DML	0.06	0.006	0.56	0.06

^a^ DML, device mass loss; LOD, limit of detection; LOQ, limit of quantitation.

**FIGURE 2:**
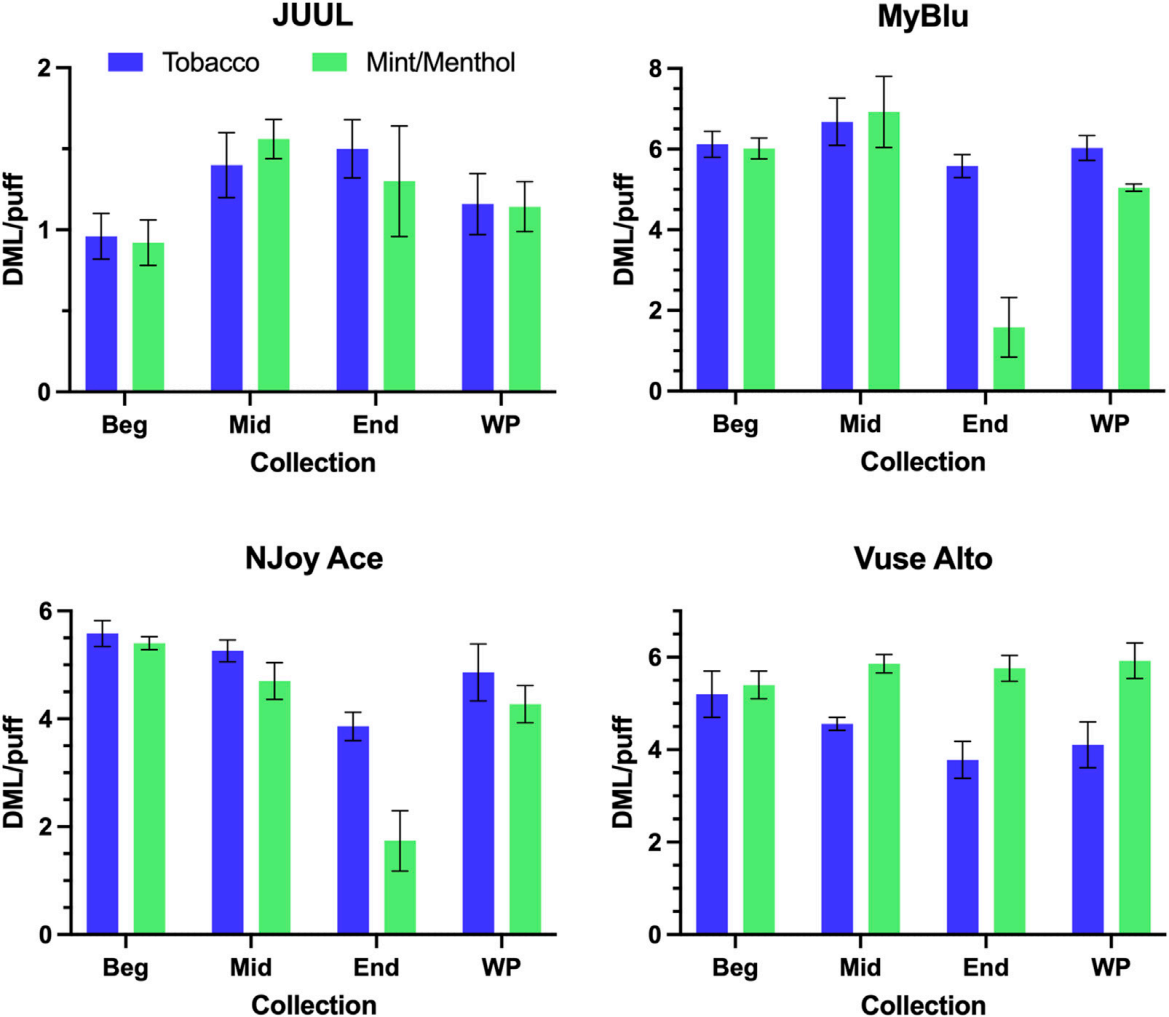
DML (mg) per puff for all products during beginning (Beg), middle (Mid), end (End), and whole pod (WP) collections.

DML per puff trends from the beginning to end puff block were unique for individual products. Some products (JUUL, MyBlu tobacco, Vuse Alto mint/menthol) were fairly consistent from beginning to end of pod life, and each 50-puff block DML per puff matched well with whole pod DML per puff (Figure 2). Two products (NJoy Ace tobacco, Vuse Alto tobacco) exhibited a slight downward trend in per puff DML over pod life, and two products (MyBlu mint/menthol and NJoy Ace mint/menthol) showed large drops (∼50%) in DML per puff in the last puff block. The relative standard deviations (RSD) in DML measurements were also higher for end puff block collections from these products compared to beginning and middle puff blocks. MyBlu, NJoy Ace and Vuse Alto products delivered whole pod DMLs in the range of 4–7 mg/puff, while the whole pod DMLs of JUUL products were around 1 mg/puff.

### 3.3 Evaluation of primary chemical constituents

**FIGURE 3:**
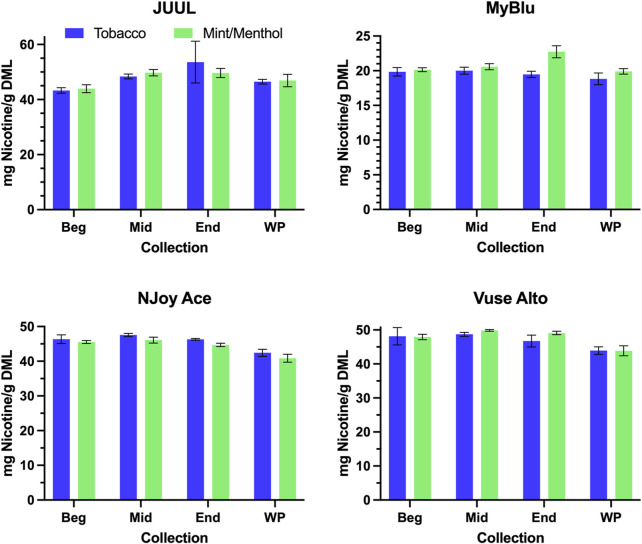
Aerosol nicotine per gram DML for beginning (Beg), middle (Mid), end (End), and whole pod (WP) collections.

When normalized to grams of DML, primary constituent measurements were fairly consistent from the beginning to end puff block for all products (Figure 3; Supplementary Table S4). For this reason, when reported per gram DML; beginning puff block and EWP yields of primary chemical constituents matched well with whole pod measurements of primary chemical constituents (Table 2; Supplementary Table S5). When normalized on a per puff basis (Supplementary Table S6), primary constituents exhibited the same trends apparent in DML per puff data from the beginning to end puff blocks (Figure 2; Supplementary Table S1), when DML yields fell approaching EOL, primary constituent measurements also decreased. Resultantly, when formatted per puff, primary chemical constituents yields measured using only the beginning puff block were higher than whole pod per puff yields for products with decreasing DML over pod life (Supplementary Table S7). EWP per puff yields, which accounted for changes in per puff yield over pod life, were more in line with whole pod per puff yields of primary constituents when changes in DML over pod life were observed. JUUL, NJoy Ace, and Vuse Alto products had whole pod nicotine yields in the range of 40–55 mg per gram of DML, while MyBlu products had around 20 mg of nicotine per gram of DML due to the nicotine strength of the products selected for the study (Figure 3).

**TABLE 2:** Nicotine yield using whole pod, EWP, and beginning puff block measurements^a^.

Product	Whole pod	EWP	Beginning
	*mg/g DML*	*mg/g DML*	*mg/g DML*
JUUL Tobacco	46.5 (0.83)	48.3 (2.7)	43.3 (0.99)
JUUL Mint/Menthol	46.9 (2.3)	47.8 (0.92)	44.0 (1.5)
MyBlu Tobacco	18.8 (0.85)	19.8 (0.45)	19.8 (0.60)
MyBlu Mint/Menthol	19.9 (0.40)	21.1 (0.26)	20.1 (0.28)
NJoy Ace Tobacco	42.4 (1.0)	46.8 (0.62)	46.4 (1.3)
NJoy Ace Mint/Menthol	40.9 (1.2)	45.4 (0.23)	45.5 (0.42)
Vuse Alto Tobacco	43.9 (1.1)	47.9 (0.92)	48.2 (2.6)
Vuse Alto Mint/Menthol	43.9 (1.5)	49.0 (0.48)	47.9 (0.79)

^a^ DML, device mass loss; EWP, extrapolated whole pod measurement.

### 3.4 Evaluation of metal chemical constituents

**FIGURE 4:**
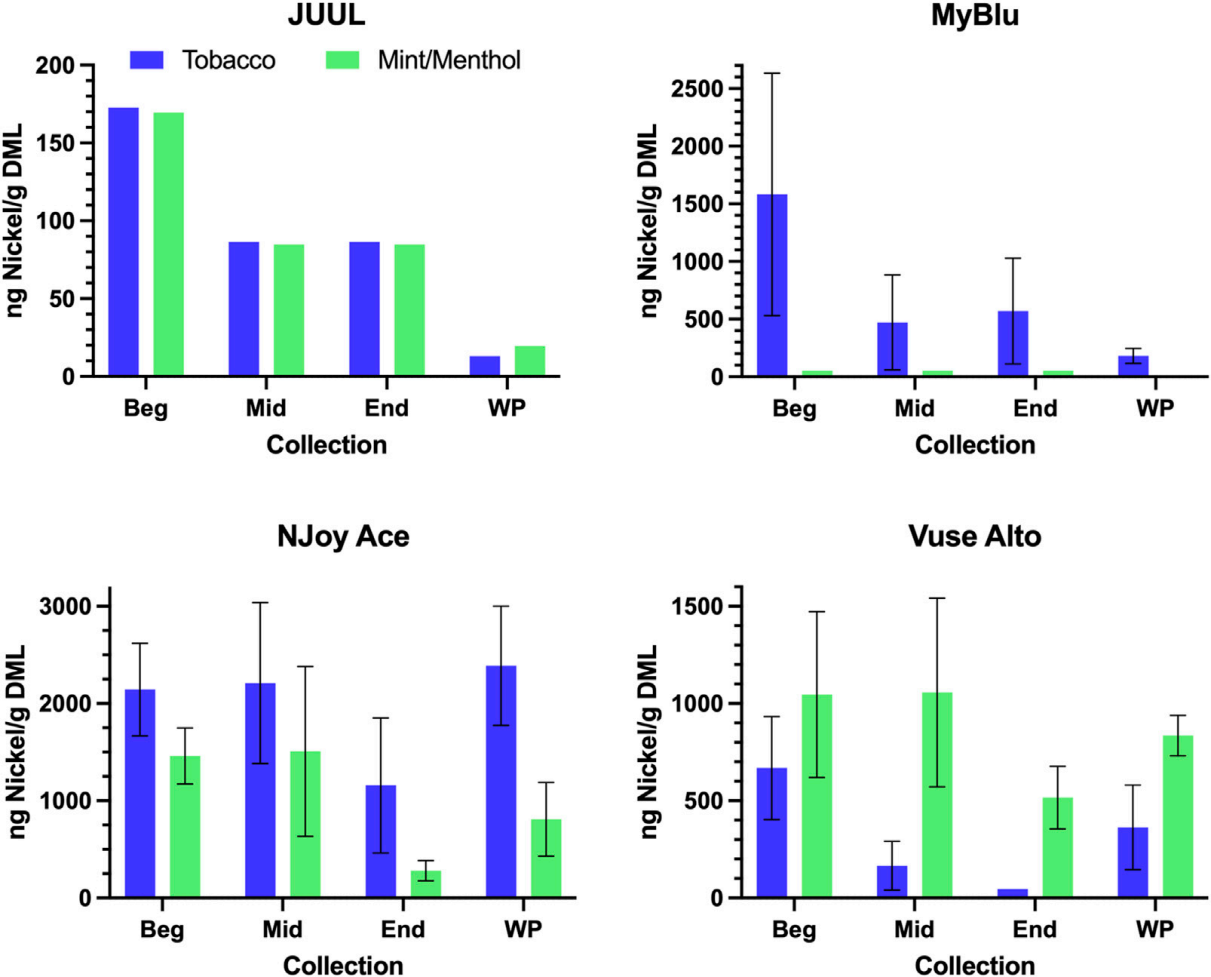
Aerosol nickel per gram DML for beginning (Beg), middle (Mid), end (End), and whole pod (WP) collection. Measurements BLOD or BLOQ are shown without error bars.

Of the 13 metals quantified in this study, nickel was the most frequently detected metal chemical constituent. Five metals (arsenic, beryllium, cadmium, cobalt, and silver) were not found above LOQ in any collections for any product. For metals detectible in 50-puff block collections, yields were frequently decreased from the beginning to the end puff block. This trend in metal chemical constituent yields was apparent, whether measurements were normalized to DML (Figure 4; Supplementary Table S8) or normalized per puff (Supplementary Table S10). Beginning puff block and EWP yields of metal chemical constituents were sometimes higher but mostly within 1 standard deviation (SD) of the whole pod yields regardless of data normalization (e.g., per gram DML or per puff) (Table 3; Supplementary Tables S9, S11). Replicate to replicate variability in metal chemical constituent measurements was high, resulting in high RSD in metal yields. Detectible metal chemical constituent yields were often less than 1000 ng/g DML, increasing the importance of method sensitivity for metal chemical constituents. There were nine instances of metal chemical constituents above LOQ in whole pod measurements but BLOQ in all beginning, middle, and end 50-puff block measurements (chromium—NJoy Ace tobacco and mint/menthol, Vuse Alto mint/menthol; iron—NJoy Ace mint/menthol, Vuse Alto mint/menthol; lead—MyBlu mint/menthol; selenium—MyBlu mint/menthol; tin—NJoy Ace tobacco and mint/menthol). Interestingly, there were also three instances of metal chemical constituents above LOQ, in 50-puff block measurement, while the corresponding whole pod measurement was BLOQ (iron—JUUL mint/menthol; lead—MyBlu tobacco; selenium—NJoy Ace mint/menthol). However, in these instances, above LOQ chemical constituent measurements had >50% RSD and were within 1 SD of LOQ.

**TABLE 3:** Nickel yield using whole pod, EWP, and beginning puff block measurements^a^.

Product	Whole pod	EWP	Beginning
	*ng/g DML*	*ng/g DML*	*ng/g DML*
JUUL Tobacco	≤13.1	≤86.4	≤173
JUUL Mint/Menthol	≤9.79	≤84.8	≤84.8
MyBlu Tobacco	180 (65)	867 (560)	1580 (1100)
MyBlu Mint/Menthol	≤10.0	≤50.3	≤50.3
NJoy Ace Tobacco	2390 (610)	1840 (540)	2140 (480)
NJoy Ace Mint/Menthol	809 (380)	973 (120)	1460 (290)
Vuse Alto Tobacco	363 (220)	287 (85)	668 (260)
Vuse Alto Mint/Menthol	836 (100)	873 (270)	1050 (430)

^a^ DML, device mass loss; EWP, extrapolated whole pod measurement.

### 3.5 Evaluation of carbonyl chemical constituents

**FIGURE 5:**
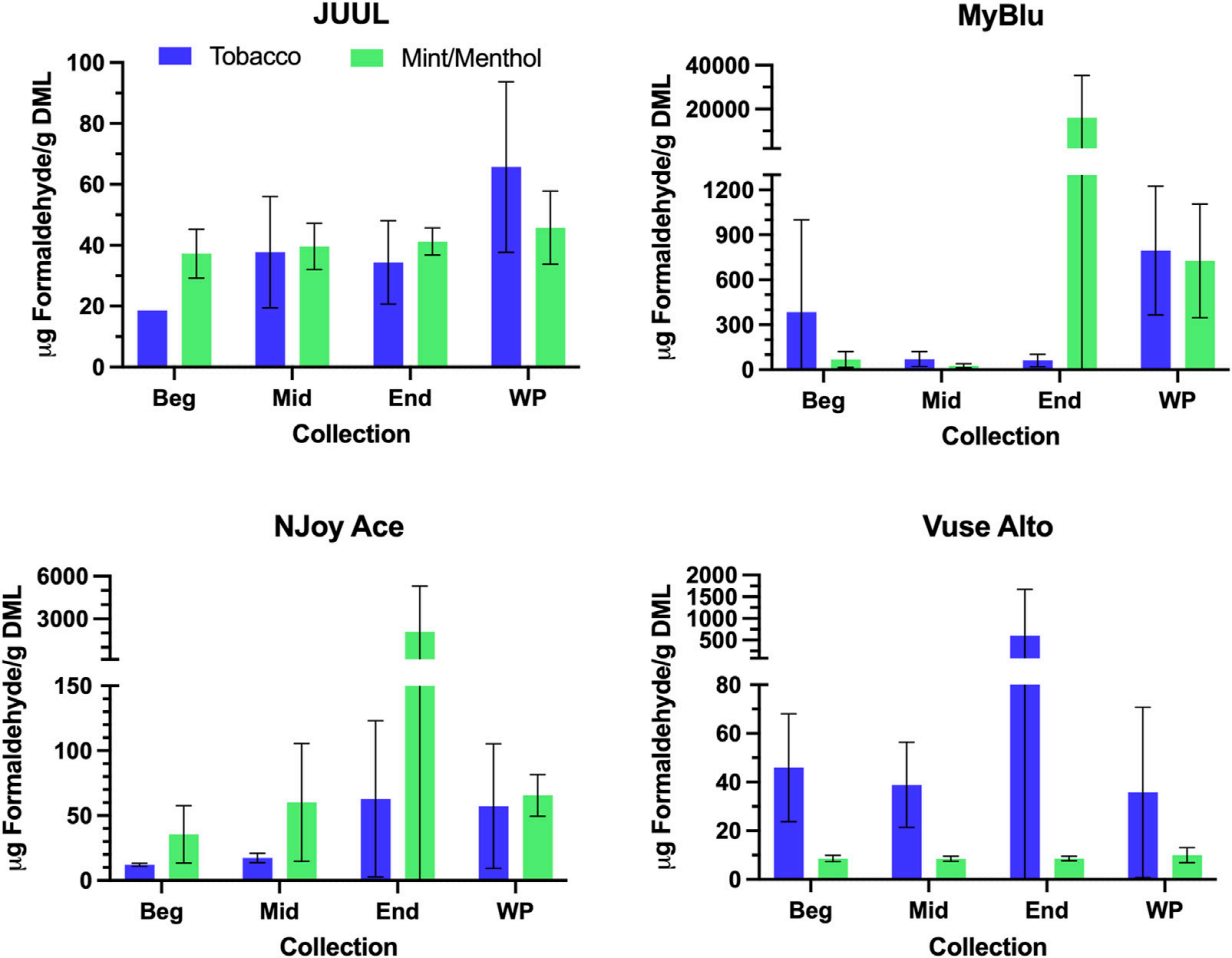
Aerosol formaldehyde per gram DML for beginning (Beg), middle (Mid), end (End), and whole pod (WP) collections. Measurements BLOD or BLOQ are shown without error bars.

**FIGURE 6:**
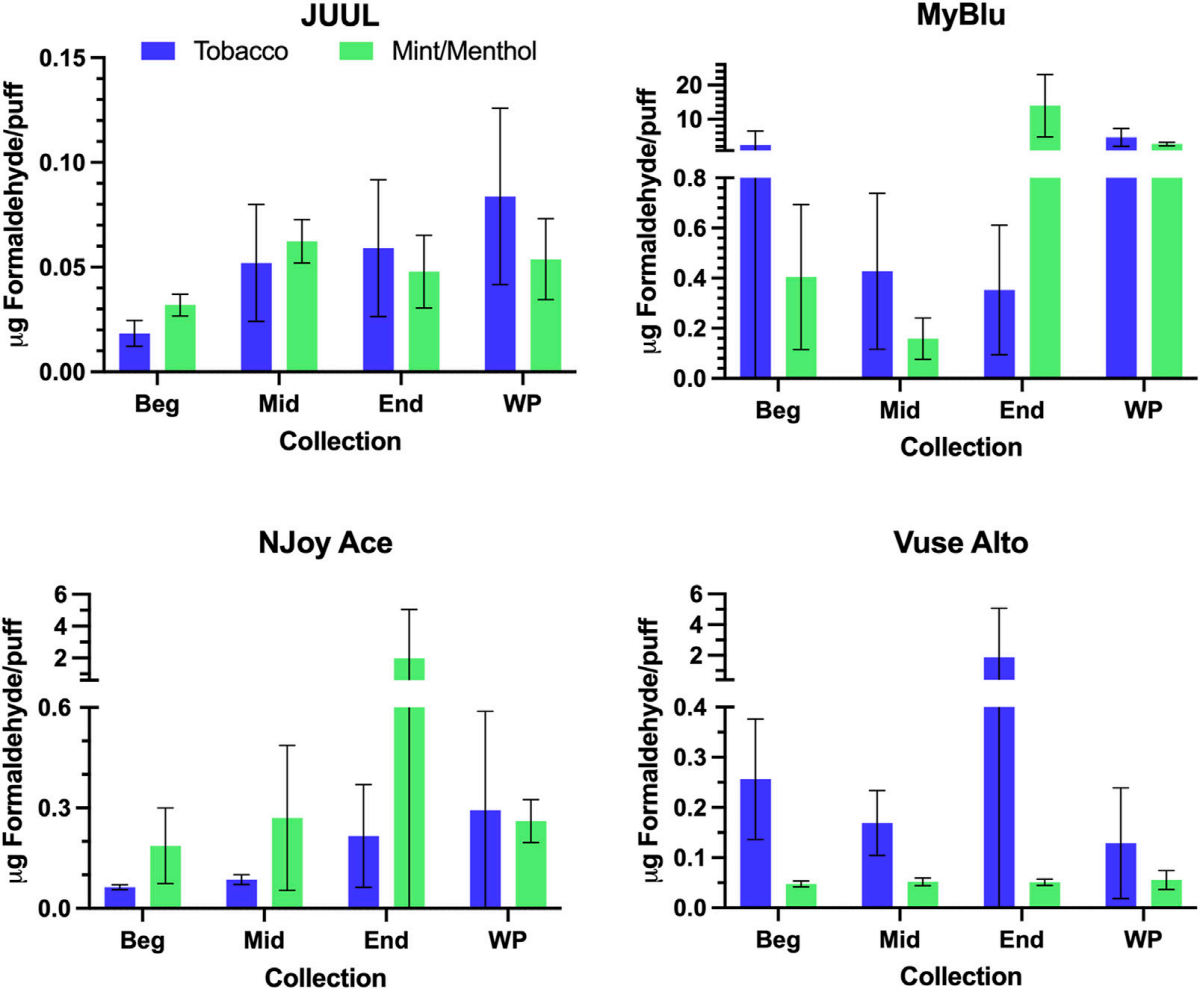
Aerosol formaldehyde per puff for beginning (Beg), middle (Mid), end (End), and whole pod (WP) collections. Measurements BLOD or BLOQ are shown without error bars.

Changes were observed in carbonyl chemical constituent yields from the beginning to the end puff block in a highly product dependent manner. Products with higher whole pod carbonyl yields (MyBlu and NJoy Ace, >200 μg/g total carbonyls, Supplementary Figure S1), sometimes had carbonyl chemical constituent measurements twenty to several hundred times higher in the end puff block than the beginning and middle puff blocks, whether normalized to DML (Figure 5; Supplemental Tables S12) or per puff (Figure 6; Supplemental Tables S14). Lower carbonyl yield products (JUUL and Vuse Alto), in general had more consistent carbonyl delivery from the beginning to the end puff block. Replicate to replicate variability in carbonyl chemical constituent measurements was extremely high for most products, resulting in whole pod, EWP and beginning puff block carbonyl yields with RSD values >50% in many cases (Table 4; Supplementary Tables S13, S15), consistent with previously published studies (Margham et al., 2016; Belushkin et al., 2020). Many carbonyl chemical constituents were BLOQ in beginning puff block and EWP yields due to higher detection limits on a per gram DML basis, precluding meaningful comparison with whole pod yields. There were 11 instances where carbonyl chemical constituents were above LOQ in whole pod chemical constituent measurements but BLOQ in all 50-puff block chemical constituent measurements (acetaldehyde—JUUL tobacco and mint/menthol; acetyl propional—MyBlu tobacco and mint/menthol, Vuse Alto mint/menthol; acrolein—JUUL tobacco and mint/menthol; n-butyraldehyde—MyBlu tobacco, Vuse Alto tobacco; crotonaldehyde–MyBlu tobacco; diacetyl–Vuse Alto mint/menthol). Due to the increasing yield of carbonyl chemical constituents over pod life, there were 21 instances where the beginning puff block chemical constituent measurements were BLOQ while whole pod chemical constituent measurements were quantifiable.

**TABLE 4:** Formaldehyde yield using whole pod, EWP, and beginning puff block measurements^a^.

Product	Whole pod	EWP	Beginning
	*µg/g DML*	*µg/g DML*	*µg/g DML*
JUUL Tobacco	65.7 (28)	30.8 (8.8)	≤18.7
JUUL Mint/Menthol	45.8 (12)	39.4 (4.8)	37.3 (8.1)
MyBlu Tobacco	795 (430)	172 (230)	384 (620)
MyBlu Mint/Menthol	530 (98)	5410 (6400)	68.6 (53)
NJoy Ace Tobacco	57.3 (48)	30.8 (21)	12.1 (1.1)
NJoy Ace Mint/Menthol	65.6 (16)	740 (1100)	35.4 (22)
Vuse Alto Tobacco	35.8 (35)	229 (360)	45.9 (22)
Vuse Alto Mint/Menthol	10.0 (3.1)	8.58 (0.95)	8.65 (1.3)

^a^ DML, device mass loss; EWP, extrapolated whole pod measurement.

For some products, formaldehyde and various other carbonyl chemical constituents increased over pod life, sometimes dramatically (Figure 5). Concurrently, DML yields per puff decreased for these same products toward the end of pod life (Figure 2). For this reason, carbonyl yields were considered per puff (Figure 6; Supplementary Tables S14, S15), as well as normalized to DML (Figure 5; Table 4; Supplementary Tables S12, S13). For carbonyls, similarly high RSD values were observed independent of normalization, and the same trends were present in carbonyl yields from the beginning to the end of pod life. Large increases in carbonyl measurements in the end puff block, particularly in combination with reduced DML and high measurement RSD (e.g., MyBlu mint/menthol, NJoy Ace mint/menthol) may indicate that dry puffing occurred in some replicates.

### 3.6 Evaluation of glycidol

**FIGURE 7:**
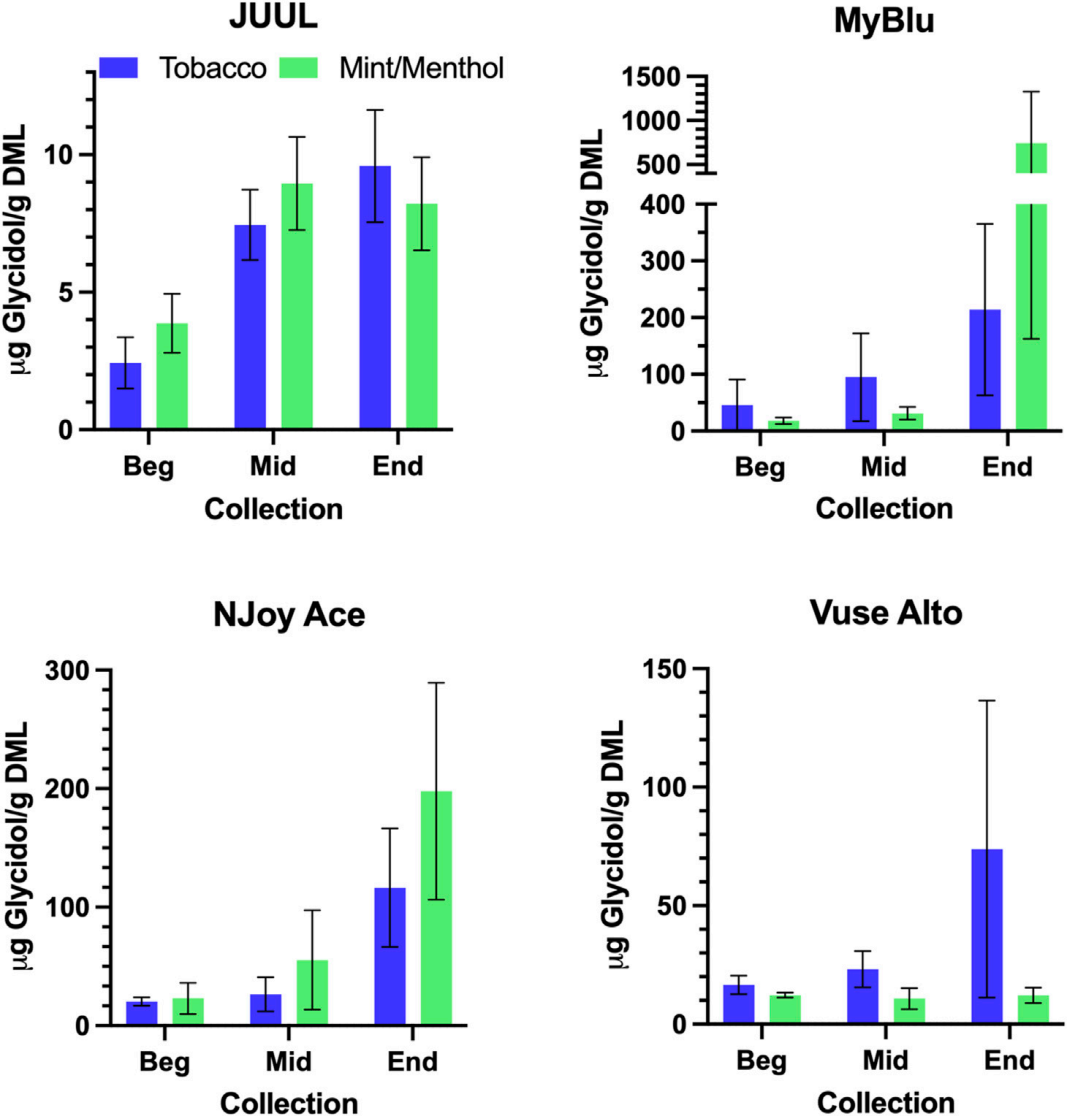
Aerosol glycidol per gram DML for beginning (Beg), middle (Mid), and end (End) collections.

Similar to carbonyl chemical constituents, glycidol yields generally increased from the beginning to end puff blocks whether normalized to DML or per puff (Figure 7; Supplementary Tables S16, S18). Due to differences in 85% EOL puff numbers between beginning, middle, and end puff block collections and corresponding glycidol whole pod collection, EWP total yields (i.e., end puff blocks) for glycidol cannot be directly compared to whole pod yields (Supplementary Table S2). Despite the increasing trend from beginning to end puff block for glycidol chemical constituent measurements for most products, beginning puff block yields per DML were within 1 SD of whole pod yields for MyBlu, NJoy Ace and Vuse Alto Menthol (Table 5; Supplementary Tables S17, S19). In some cases likely due to high levels of variability in yield measurements.

**TABLE 5:** Glycidol yield using whole pod and beginning puff block measurements^a^.

Product	Whole pod	Beginning
	*µg/g DML*	*µg/g DML*
JUUL Tobacco	4.95 (0.91)	1.20 (0.94)
JUUL Mint/Menthol	5.03 (0.89)	2.94 (0.33)
MyBlu Tobacco	31.6 (20)	45.4 (46)
MyBlu Mint/Menthol	18.2 (1.7)	18.1 (5.7)
NJoy Ace Tobacco	16.9 (4.7)	20.4 (3.4)
NJoy Ace Mint/Menthol	9.77 (2.2)	23.1 (13)
Vuse Alto Tobacco	89.7 (28)	16.6 (3.9)
Vuse Alto Mint/Menthol	45.7 (46)	12.2 (1.1)

^a^ DML, device mass loss.

## 4 Discussion

Total primary constituent yields were accurately determined by the EWP collection strategy when normalized to DML, provided analyte concentrations were above LOQ. This was also true for a collection strategy consisting of only the beginning 50-puffs. However, because per puff DML was inconsistent for some products, beginning 50-puff primary constituent yields were higher than whole pod yields for those products with inconsistent DML per puff when data was normalized per puff. EWP yields of primary constituents per puff matched well with whole pod per puff yields, even when changes in DML were observed over pod life. Metal chemical constituent yields frequently decreased over pod life, whether normalized to DML or per puff. Due to low metals concentrations and variability in metal yields, whole pod collection was the most viable strategy for accurately determining the yields of metals. However, beginning puff block or multiple collection methodologies could be appropriate for estimating metal chemical constituent yields in some products if the collection methods are properly validated and appropriately sensitive. Carbonyl and glycidol yields frequently increased over pod life, in some cases dramatically. Beginning puff collections, even those incorporating a large fraction of total aerosol, are likely to under-report true carbonyl and glycidol chemical constituent yields. To accurately report yields of carbonyls and glycidol care should be taken in designing an appropriate collection strategy to test user relevant puffing conditions over the whole pod life while limiting dry puffing. Whole pod collections improved the accuracy and sensitivity of metal, carbonyl, and glycidol yield determinations in the aerosol of ENDS products compared to smaller aerosol collections due to the dynamic nature of those chemical constituents’ yields over the life of a single pod.

Due to the low yields of many chemical constituents in ENDS products, analytical method sensitivity can be an important factor in accurately reporting yields (Vu et al., 2015). Increasing the collected aerosol mass can improve method detection limits by increasing the concentration of all analytes in an extract solution, provided the mass of aerosol collected does not exceed the limit of solubility for solvent extraction. Previously published research has reported aerosol collections ranging from 15 to 150 puffs (Calafat et al., 2004; Cheng, 2014; Vu et al., 2015; Flora et al., 2017; Chen et al., 2021; Soleimani et al., 2022). For the products examined here, compared to collection of a single 50-puff block, collection of whole pod aerosol constituted an increase of roughly 500%–1000% of total collected mass. As a result, whole pod measurements were more frequently above LOQ compared to beginning, middle, and end puff block-based measurements. Of the 208 chemical constituent measurements (eight products, 26 chemical constituents) 113 were above LOQ for whole pod measurements, compared to 94 above LOQ measurements in one or more of the beginning, middle, and end puff blocks, and only 85 above LOQ in the beginning puff block alone. Conversely, for three metal chemical constituent measurements, whole pod measurements were BLOQ while one or more 50-puff block measurement was above LOQ. All of these measurements were within 1 SD of LOQ, suggesting pod-to-pod variability in metal yields may explain this inconsistency.

The increased sensitivity afforded by this methodology allows a more accurate comparison of these ENDS products with yields from CC reported in the literature. All products tested exhibited large reductions in carbonyls, with the grand mean of all whole pod collections producing approximately 4% of formaldehyde found in the 3R4F reference cigarette when compared on a per puff basis (Supplementary Table S15) (Jaccard et al., 2019). The product with the highest formaldehyde under whole pod collection, MyBlu tobacco, yielded only 20% of that of 3R4F when compared on a per puff basis. Similarly, the highest acetaldehyde from the whole pod collections in this study (MyBlu tobacco) was 0.3% of 3R4F and the grand mean of all products tested was 0.1% of 3R4F reference cigarette when compared on a per puff basis. All other measured carbonyls were similarly reduced compared to 3R4F. However, some ENDS products had increased metals yields (Supplementary Table S11) in comparison to 3R4F smoke. For nickel, Pappas et al. (2014) reported non-detectable values for 3R4F (<0.38 ng/stick). In the current study, JUUL tobacco (<0.013 ng/puff), JUUL mint/menthol (<0.011 ng/puff), and MyBlu mint/menthol (<0.015 ng/puff) were also BLOD. The five other ENDS products yielded an average of 4.7 ng/puff, with the highest measured nickel in the NJoy Ace tobacco at 12 ng/puff. Lead was also quantifiable in the NJoy Ace tobacco (2.8 ng/puff) and Vuse Alto tobacco (1.4 ng/puff) at levels comparable to or higher than the 3R4F reference cigarette (9.2 ng/stick, using 10 puffs/stick). As, Cd, and Co were BLOD or BLOQ for all ENDS in the current study, but reported to be present in 3R4F smoke at 2.9 ng/stick (As), 39 ng/stick (Cd), and 0.049 ng/stick (Co) (Pappas et al., 2014). For glycidol the tested ENDS products ranged from 0.006 µg/puff (JUUL tobacco and mint/menthol) to 0.38 µg/puff (Vuse Alto tobacco) under ISO 20768 conditions (Supplementary Table S19). Limited data has been published on the levels of glycidol in the 3R4F reference cigarette, only one value (1.76 µg/stick), collected under Health Canada Intense puffing, was available for comparison (St Helen et al., 2018). The whole pod collections of six out of eight ENDS products tested had lower per puff glycidol emissions compared to 3R4F when compared on a per puff basis.

Selecting the number of puffs for an appropriate whole pod collection when testing multiple products is important and can be difficult due to the differences in pod and device design characteristics. This is a limitation of EOL based collection strategies. This study used a uniform set point of 85% of EOL for all products. Total DML at EOL was determined using aerosol collections of 50-puff blocks that ceased when two consecutive collections were <10 mg DML. The relatively high RSD observed in some end puff block DML measurements (Supplementary Tables S1, S2), coupled with high end puff block carbonyl yields for those products indicates that a DML cutoff >10 mg/50 puffs may be appropriate for some products. EOL determination was performed twice for MyBlu, NJoy Ace, and Vuse Alto due to separate sample collections for whole pod glycidol measurements, resulting in different whole pod puff numbers. EOL also varied between brands and flavors. High aerosol yield products may be better suited to 10 or 20 puff collections for more precise EOL determination, and products with highly consistent per puff yields may be capable of EOL set points of 90+%. During aerosol sample collection two mint/menthol products (MyBlu and NJoy Ace) had end puff block DMLs around 50% lower than beginning and middle blocks for primary constituents, metals, and carbonyl collections. These large drops in DML were not observed in tobacco formulations, possibly suggesting the need for formulation specific collection parameters. The levels of carbonyls detected in the MyBlu mint/menthol end puff block were above the levels of carbonyls reported to correlate with end user detection of “dry puff flavor” in ENDS aerosol (Visser et al., 2021). NJoy Ace mint/menthol and Vuse Alto tobacco exhibited large increases in carbonyl yields in the end puff block with >100% RSD. Unlike MyBlu carbonyls yields, these increased end puff block yields were not reflected in whole pod measurements. Despite both products having below 15% RSD in whole pod DML (the basis for ending collections at 85% EOL), these results suggest dry puffing may have occurred in some replicates. These results also highlight that the analysis of end puff blocks may overestimate the HPHC yield from the product, while a single collection from puff 1%–85% EOL will give a more accurate overall HPHC yield since the impact of the end puffs will be minimized by the collection of a large number of puffs from the device. However, this collection methodology may not be appropriate for all ENDS device types, i.e., devices with large capacity and high aerosol yields. In these cases, multiple large (∼0.5–1 g) collections at intervals over pod life may be more appropriate. Additional work may be needed to determine appropriate experimental procedures to collect a whole pod aerosol sample while avoiding dry puffing conditions for all products (Farsalinos et al., 2015).

While primary constituent per DML measurements were consistent over pod life, per puff measurements of primary constituents decrease in the end puff block for some products, consistent with observed trends in DML. Previous research has shown changes in DML occur over ENDS pod life (Gupta et al., 2021), but we are not aware of studies reporting the yields of primary constituents in discrete blocks over product life. Previous studies have reported high variability in metal yields between product categories, brands, flavors and other factors (Zhao et al., 2019; Gray et al., 2020; Kapiamba et al., 2022). Rastian et al. (2022) reported increasing concentrations of chromium, nickel, copper, and lead over four consecutive 10 puff blocks in the aerosol from a single high power open system ENDS device. However, the methodology used in the above studies is not likely relevant to user exposure or comparison with low power closed system ENDS (Soulet and Sussman, 2022a; Soulet and Sussman, 2022b). In the current study, metal yields generally decreased over pod life. Previous research has already suggested carbonyl yields from ENDS can increase from the initial puffs to the end of pod life (Guthery, 2016; Farsalinos and Gillman, 2018; Belushkin et al., 2020), and large values for carbonyl emissions from ENDS are thought to come predominantly from dry puffing conditions, which may not be relevant to user exposure (Farsalinos et al., 2015; Geiss et al., 2016; Farsalinos and Gillman, 2018; Soulet and Sussman, 2022a). Yields of carbonyl chemical constituents also changed over pod life in the current study, most often increasing from beginning to end of pod life, in agreement with previous studies (Belushkin et al., 2020). Despite aerosol collection methodology designed to limit dry puffing, one product in the current study (MyBlu mint/menthol) generated carbonyl emissions in the end puff block at a level previously reported to be perceptible to end users (Visser et al., 2021). Glycidol in open system ENDS aerosol has been shown to increase at high power settings (Uchiyama et al., 2020), but yields over closed system pod life have not been studied. Similar to carbonyl chemical constituents, glycidol yields increased over pod life for the majority of products included in this study.

In conclusion, whole pod aerosol collection significantly increased the sensitivity of aerosol chemical constituent measurements for low concentration constituents (i.e., non-primary constituents). For the primary constituent levels of propylene glycol, glycerin, nicotine, and menthol, acceptable accuracy in yield determination was observed using non-whole pod collection strategies that account for changing DML. DML independent changes in chemical constituent yields were observed over pod life for selected metals, carbonyls and glycidol, in a range of closed pod-based ENDS products. Any aerosol sampling strategy attempting to address total chemical constituent yields must account for potential changes in yields over pod life. As such, beginning puff block collection strategies are inappropriate for some analytes and products. Due to differences in chemical constituent yield patterns between products, aerosol collection strategies must be verified for each specific product and chemical constituent in question. Collection strategies that account for yields close to EOL without collecting whole pod aerosol may be appropriate for some products and analytes that have consistent yield patterns over pod life and do not benefit from the increased sensitivity of whole pod collection. Whole pod collection remains the most effective strategy for accounting for total chemical constituent yields. Regulatory agencies, including FDA, should issue guidance on ENDS aerosol collection methods relating to partial and whole pod collections when chemical constituents of interest are variable over product lifetime.

## Data Availability

The original contributions presented in the study are included in the article/Supplementary Material, further inquiries can be directed to the corresponding author.

## Abbreviations

ENDS, electronic nicotine delivery systems; PMTA, premarket tobacco product application; DML, device mass loss; CC, combustible cigarette; EOL, end of life; Beg, beginning puff block measurement; Mid, middle puff block measurement; End, end puff block measurement; EWP, estimated Whole Pod measurement; WP, whole pod measurement; LOD, limit of detection; BLOD, below limit of detection; LOQ, limit of quantitation; BLOQ, below limit of quantitation; Meas, measurement; SD, standard deviation; RSD, relative standard deviation.
