# Supplementary figures and images for: Determination of chemical constituent yields in e-cigarette aerosol using partial and whole pod collections, a comparative analysis
Source: Front Chem. 2023 Sep 7;11:1223967. doi: 10.3389/fchem.2023.1223967 (PMC10512464; doi:10.3389/fchem.2023.1223967)

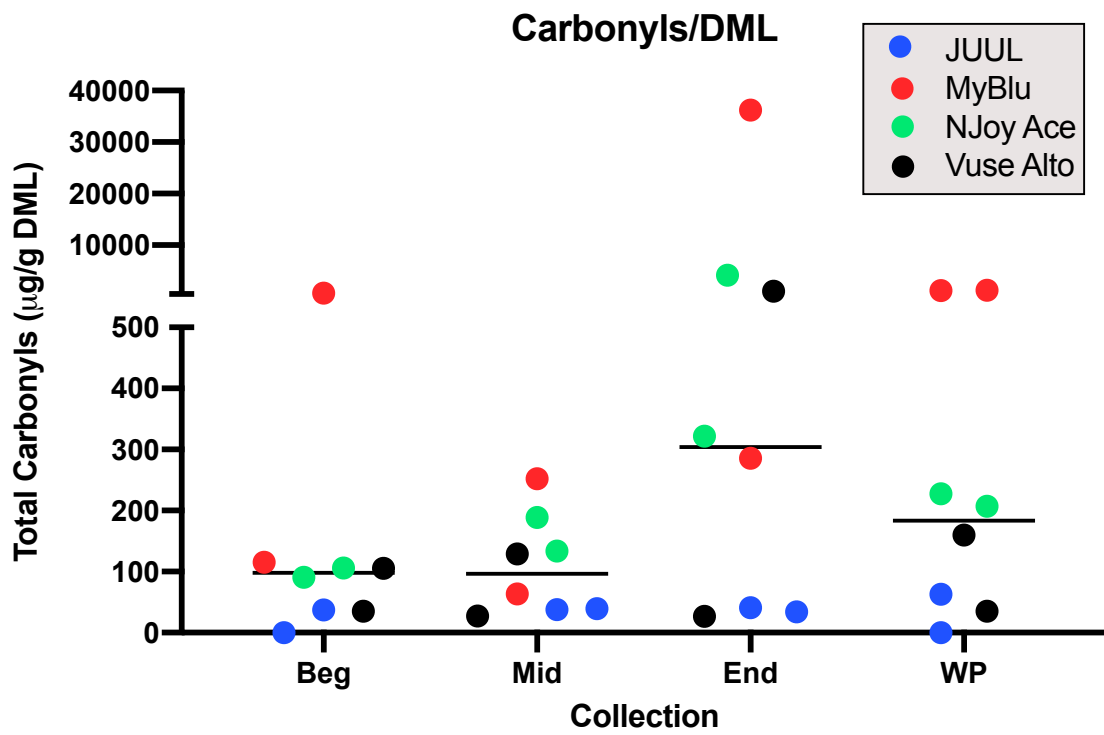

Supplement: Supplementary file 1 [file DataSheet2.PDF]
