# Supplementary material for: Determination of chemical constituent yields in e-cigarette aerosol using partial and whole pod collections, a comparative analysis
Source: Front Chem. 2023 Sep 7;11:1223967. doi: 10.3389/fchem.2023.1223967 (PMC10512464; doi:10.3389/fchem.2023.1223967)
Supplement: Supplementary file 2 [file DataSheet1.PDF]

Table S1. Puff counts, mean DML, and standard deviation for beginning, middle, and end puff block, and whole pod primary constituents, metals and carbonyl measurements of aerosol from eight closed system ends products.

	WP			Beginning			Middle			End			Total of B/M/E		
**Product**	**Puff Count**	**DML (g)**	**Std Dev**	**Puff Block**	**DML (g)**	**Std Dev**	**Puff Block**	**DML (g)**	**Std Dev**	**Puff Block**	**DML (g)**	**Std Dev**	**Puff Count**	**DML (g)**	**Std Dev**
**JUUL Tobacco**	371	0.43	0.07	1-50	0.048	0.007	191-240	0.070	0.010	322-371	0.075	0.009	150	0.193	0.01
**JUUL Mint/Menthol**	455	0.52	0.07	1-50	0.046	0.007	264-313	0.078	0.006	406-455	0.065	0.017	150	0.189	0.01
**myBlu Tobacco**	262	1.58	0.08	1-50	0.306	0.016	107-156	0.334	0.029	213-262	0.279	0.014	150	0.919	0.15
**myBlu Mint/Menthol**	323	1.63	0.03	1-50	0.301	0.013	116-165	0.346	0.044	274-323	0.079	0.037	150	0.726	0.05
**NJOY Ace Tobacco**	360	1.75	0.19	1-50	0.279	0.012	159-208	0.263	0.010	311-360	0.193	0.013	150	0.735	0.01
**NJOY Ace Mint/Menthol**	405	1.73	0.14	1-50	0.270	0.006	169-218	0.235	0.017	356-405	0.087	0.028	150	0.592	0.02
**Vuse Alto Tobacco**	363	1.49	0.18	1-50	0.260	0.025	148-197	0.228	0.007	314-363	0.189	0.020	150	0.677	0.03
**Vuse Alto Mint/Menthol**	260	1.54	0.10	1-50	0.271	0.015	108-157	0.293	0.010	211-260	0.288	0.014	150	0.852	0.02

Note: WP = Whole pod. B/M/E = Beginning, Middle, and End puff segments. DML = Device mass loss. EOL = End of life. Std Dev = Standard deviation. Results for all collections excluding glycidol.

Table S2. Puff counts, mean DML, and standard deviation for beginning, middle, and end puff block, and whole pod glycidol measurements of aerosol from eight closed system ends products.

	WP			Beginning			Middle			End			Total of B/M/E		
**Product**	**Puff Count**	**DML (g)**	**Std Dev**	**Puff Block**	**DML (g)**	**Std Dev**	**Puff Block**	**DML (g)**	**Std Dev**	**Puff Block**	**DML (g)**	**Std Dev**	**Puff Count**	**DML (g)**	**Std Dev**
**JUUL Tobacco**	371	0.44	0.09	1–50	0.031	0.020	191-240	0.065	0.017	322-371	0.069	0.022	150	0.165	0.025
**JUUL Mint/Menthol**	455	0.55	0.06	1–50	0.050	0.018	264-313	0.073	0.014	406-455	0.053	0.034	150	0.176	0.022
**myBlu Tobacco**	284	1.42	0.09	1–50	0.263	0.049	107-156	0.305	0.020	213-262	0.221	0.087	150	0.789	0.065
**myBlu Mint/Menthol**	263	1.44	0.07	1–50	0.289	0.025	116-165	0.328	0.082	274-323	0.042	0.039	150	0.659	0.118
**NJOY Ace Tobacco**	289	1.79	0.08	1–50	0.264	0.006	159-208	0.251	0.012	311-360	0.168	0.030	150	0.683	0.047
**NJOY Ace Mint/Menthol**	292	1.81	0.05	1–50	0.247	0.020	169-218	0.208	0.054	356-405	0.136	0.027	150	0.591	0.056
**Vuse Alto Tobacco**	314	1.37	0.22	1–50	0.272	0.009	148-197	0.231	0.021	314-363	0.181	0.027	150	0.684	0.043
**Vuse Alto Mint/Menthol**	262	1.23	0.26	1–50	0.276	0.008	108-157	0.280	0.008	211-260	0.270	0.026	150	0.826	0.016

Note: WP = Whole pod. B/M/E = Beginning, Middle, and End puff segments. DML = Device mass loss. EOL = End of life. Std Dev = Standard deviation. Glycidol results only.

Table S3. Mean detection limits of all aerosol constituent measurements for each analytical method using puff block and WP collections

			LOD		LOQ	
**Method**	**Units**	**Chemical Constituent**	**Puff Block**	**WP**	**Puff Block**	**WP**
Primary Constituents	mg/collection	Glycerol	0.19	5.03	1.95	15.09
		Menthol	0.12	0.19	1.23	0.58
		Nicotine	0.03	0.60	0.16	1.80
		Propylene Glycol	0.16	1.60	1.61	4.80
		Water	0.78	4.04	2.57	12.11
Metals	ng/collection	Arsenic	10.0	10.0	50.0	50.0
		Beryllium	2.20	2.20	20.0	20.0
		Cadmium	1.00	1.00	5.00	5.00
		Chromium	10.0	10.0	50.0	50.0
		Cobalt	0.50	0.50	5.00	5.00
		Copper	25.0	25.0	75.0	75.0
		Iron	100	100	200	200
		Lead	2.50	2.50	10.0	10.0
		Nickel	5.00	5.00	10.0	10.0
		Selenium	3.90	3.90	20.0	20.0
		Silver	1.00	1.00	5.00	5.00
		Tin	10.0	10.0	50.0	50.0
		Zinc	169	394	555	1295
Carbonyls	μg/collection	Acetaldehyde	0.17	0.17	1.13	1.13
		Acetyl Propionyl	0.11	0.11	1.13	1.13
		Acrolein	0.11	0.11	1.13	1.13
		n-Butyraldehyde	0.12	0.12	1.13	1.13
		Crotonaldehyde	0.11	0.11	1.12	1.12
		Diacetyl	0.12	0.12	1.15	1.15
		Formaldehyde	0.25	0.25	1.13	1.13
Glycidol	μg/collection	Glycidol	0.01	0.01	0.06	0.06

Note: WP = Whole Pod. LOD = Limit of detection. LOQ = Limit of quantitation.

Table S4. Mean and standard deviation of primary chemical constituent measurements per g DML in aerosol from eight closed system ends products during beginning, middle, and end puff blocks.

Product	Puff Block	Glycerol	Menthol	Nicotine	Propylene Glycol	Water
		mg/g DML	*mg/g DML*	*mg/g DML*	*mg/g DML*	*mg/g DML*
**JUUL Tobacco**	**Beg**	598.71 (15.02)	BLOD	43.28 (0.99)	198.33 (3.08)	178.59 (38.80)
	**Mid**	638.64 (9.28)	BLOD	48.40 (0.88)	239.47 (9.67)	144.58 (37.37)
	**End**	624.70 (27.10)	BLOD	53.60 (7.61)	297.56 (68.28)	152.65 (34.75)
**JUUL Mint/Menthol**	**Beg**	593.00 (47.05)	BLOQ	43.96 (1.45)	202.62 (10.17)	165.04 (3.32)
	**Mid**	640.24 (12.31)	BLOQ	49.73 (1.16)	251.57 (10.06)	118.85 (7.87)
	**End**	558.42 (40.69)	BLOQ	49.63 (1.69)	295.33 (32.51)	177.44 (58.85)
**myBlu Tobacco**	**Beg**	514.54 (27.98)	BLOD	19.84 (0.60)	317.07 (9.47)	135.37 (8.51)
	**Mid**	532.87 (11.28)	BLOD	20.01 (0.51)	344.75 (13.33)	146.52 (45.75)
	**End**	496.96 (10.77)	BLOD	19.47 (0.45)	396.98 (14.72)	129.72 (6.97)
**myBlu Mint/Menthol**	**Beg**	619.96 (2.82)	BLOQ	20.14 (0.28)	248.33 (6.66)	90.51 (3.22)
	**Mid**	637.23 (7.93)	BLOQ	20.57 (0.44)	262.52 (9.50)	92.78 (3.28)
	**End**	460.98 (56.15)	BLOQ	22.73 (0.86)	314.73 (22.78)	221.98 (14.41)
**NJOY Ace Tobacco**	**Beg**	498.65 (13.87)	BLOD	46.36 (1.26)	293.92 (6.90)	131.57 (4.08)
	**Mid**	493.22 (7.31)	BLOD	47.58 (0.46)	342.00 (4.23)	124.46 (8.10)
	**End**	470.36 (12.16)	BLOD	46.31 (0.24)	362.04 (18.89)	132.81 (12.80)
**NJOY Ace Mint/Menthol**	**Beg**	486.87 (6.69)	BLOQ	45.54 (0.42)	303.43 (7.19)	156.66 (10.91)
	**Mid**	457.89 (6.86)	BLOQ	46.09 (0.86)	338.67 (13.88)	156.28 (11.66)
	**End**	432.34 (17.15)	BLOQ	44.7 (0.47)	354.25 (19.26)	171.66 (22.20)
**Vuse Alto Tobacco**	**Beg**	500.87 (15.92)	BLOD	48.15 (2.55)	324.25 (13.47)	135.30 (11.53)
	**Mid**	473.34 (10.87)	BLOD	48.71 (0.56)	384.03 (12.68)	120.29 (5.88)
	**End**	440.71 (26.36)	BLOD	46.73 (1.76)	399.68 (25.33)	138.22 (14.25)
**Vuse Alto Mint/Menthol**	**Beg**	516.96 (9.45)	5.95 (1.59)	47.92 (0.79)	305.02 (9.46)	117.9 (14.87)
	**Mid**	501.54 (10.21)	8.69 (0.27)	49.85 (0.25)	349.41 (7.20)	101.62 (13.99)
	**End**	467.46 (11.35)	9.63 (0.46)	49.10 (0.52)	387.76 (9.87)	106.84 (15.62)

Note: DML = Device mass loss. Beg = Beginning puff block. Mid = Middle puff block. End = End puff block. BLOD = Below limit of detection. BLOQ = Below limit of quantitation.

Table S5. Mean and standard deviation of primary chemical constituent yields per g DML in aerosol from eight closed system ends products using whole pod, EWP, and beginning puff block measurements.

Product	Measurement	Glycerol	Menthol	Nicotine	Propylene Glycol	Water
		mg/g DML	*mg/g DML*	*mg/g DML*	*mg/g DML*	*mg/g DML*
**JUUL Tobacco**	**WP**	604 (17)	BLOD	46.5 (0.83)	231 (3.5)	121 (7.3)
	**EWP**	622 (13)	BLOD	48.3 (2.7)	244 (25)	163 (35)
	**Beg**	599 (15)	BLOD	43.3 (0.99)	198 (3.1)	179 (39)
**JUUL Mint/Menthol**	**WP**	577 (30)	7.34 (0.26)	46.9 (2.3)	234 (9.2)	123 (14)
	**EWP**	593 (12)	BLOQ	47.8 (0.92)	247 (8.7)	156 (19)
	**Beg**	593 (47)	BLOQ	44.0 (1.5)	203 (10)	165 (3.3)
**myBlu Tobacco**	**WP**	475 (21)	BLOD	18.8 (0.85)	328 (27)	95.1 (3.5)
	**EWP**	515 (13)	BLOD	19.8 (0.45)	353 (10)	137 (18)
	**Beg**	515 (28)	BLOD	19.8 (0.60)	317 (9.5)	135 (8.5)
**myBlu Mint/Menthol**	**WP**	569 (13)	5.69 (0.15)	19.9 (0.40)	254 (4.1)	91.1 (3.6)
	**EWP**	573 (17)	BLOQ	21.1 (0.26)	275 (6.8)	136 (4.4)
	**Beg**	620 (2.8)	BLOQ	20.1 (0.28)	248 (6.7)	90.5 (3.2)
**NJOY Ace Tobacco**	**WP**	416 (8.0)	BLOD	42.4 (1.0)	284 (10)	132 (5.8)
	**EWP**	487 (5.9)	BLOD	46.8 (0.62)	333 (7.4)	130 (5.7)
	**Beg**	499 (14)	BLOD	46.4 (1.3)	294 (6.9)	132 (4.1)
**NJOY Ace Mint/Menthol**	**WP**	407 (14)	2.10 (0.08)	40.9 (1.2)	286 (7.3)	128 (10)
	**EWP**	459 (5.1)	BLOQ	45.4 (0.23)	332 (9.4)	162 (14)
	**Beg**	487 (6.7)	BLOQ	45.5 (0.42)	303 (7.2)	157 (11)
**Vuse Alto Tobacco**	**WP**	428 (17)	BLOD	43.9 (1.1)	321 (8.1)	111 (13)
	**EWP**	472 (9.7)	BLOD	47.9 (0.92)	369 (15)	131 (4.7)
	**Beg**	501 (16)	BLOD	48.2 (2.6)	324 (13)	135 (12)
**Vuse Alto Mint/Menthol**	**WP**	426 (18)	8.32 (0.21)	43.9 (1.5)	308 (6.9)	84.7 (5.5)
	**EWP**	495 (8.1)	8.09 (0.64)	49.0 (0.48)	347 (3.6)	109 (15)
	**Beg**	517 (9.5)	5.95 (1.6)	47.9 (0.79)	305 (9.5)	118 (15)

Note: DML = Device mass loss. WP = Whole pod measurement. EWP = Extrapolated whole pod measurement. Beg = Beginning puff block. BLOD = Below Limit of detection. BLOQ = Below Limit of quantitation.

Table S6. Mean and standard deviation of primary chemical constituent measurements per puff in aerosol from eight closed system ends products during beginning, middle, and end puff blocks.

Product	Puff Block	Glycerol	Menthol	Nicotine	Propylene Glycol	Water
		mg/puff	*mg/puff*	*mg/puff*	*mg/puff*	*mg/puff*
**JUUL Tobacco**	**Beg**	0.76 (0.16)	BLOD	0.055 (0.013)	0.25 (0.06)	0.20 (0.01)
	**Mid**	1.17 (0.19)	BLOD	0.088 (0.015)	0.44 (0.08)	0.21 (0.04)
	**End**	0.97 (0.57)	BLOD	0.080 (0.046)	0.42 (0.24)	0.17 (0.04)
**JUUL Mint/Menthol**	**Beg**	0.72 (0.18)	BLOQ	0.053 (0.010)	0.24 (0.05)	0.20 (0.02)
	**Mid**	1.05 (0.26)	BLOQ	0.082 (0.020)	0.42 (0.12)	0.24 (0.02)
	**End**	0.77 (0.47)	BLOQ	0.066 (0.037)	0.38 (0.20)	0.10 (0.08)
**myBlu Tobacco**	**Beg**	3.12 (0.27)	BLOD	0.12 (0.01)	1.92 (0.12)	0.88 (0.03)
	**Mid**	3.18 (0.19)	BLOD	0.12 (0.01)	2.06 (0.06)	0.91 (0.31)
	**End**	3.03 (0.07)	BLOD	0.12 (0.004)	2.42 (0.15)	0.78 (0.09)
**myBlu Mint/Menthol**	**Beg**	3.40 (0.26)	BLOQ	0.11 (0.01)	1.36 (0.12)	0.59 (0.03)
	**Mid**	3.69 (0.18)	BLOQ	0.12 (0.004)	1.52 (0.05)	0.58 (0.03)
	**End**	1.05 (0.50)	BLOQ	0.050 (0.017)	0.70 (0.23)	0.13 (0.12)
**NJOY Ace Tobacco**	**Beg**	2.92 (0.15)	BLOD	0.27 (0.01)	1.72 (0.09)	0.78 (0.07)
	**Mid**	2.52 (0.20)	BLOD	0.24 (0.02)	1.75 (0.11)	0.64 (0.14)
	**End**	2.04 (0.12)	BLOD	0.20 (0.01)	1.57 (0.16)	0.42 (0.14)
**NJOY Ace Mint/Menthol**	**Beg**	2.67 (0.12)	BLOQ	0.25 (0.02)	1.67 (0.12)	0.84 (0.04)
	**Mid**	1.91 (0.37)	BLOQ	0.19 (0.04)	1.42 (0.29)	0.64 (0.08)
	**End**	1.11 (0.47)	BLOQ	0.12 (0.05)	0.92 (0.44)	0.48 (0.06)
**Vuse Alto Tobacco**	**Beg**	2.92 (0.25)	BLOD	0.28 (0.03)	1.89 (0.17)	0.73 (0.06)
	**Mid**	2.30 (0.38)	BLOD	0.24 (0.04)	1.87 (0.35)	0.53 (0.05)
	**End**	1.35 (0.59)	BLOD	0.14 (0.06)	1.18 (0.39)	0.47 (0.04)
**Vuse Alto Mint/Menthol**	**Beg**	2.99 (0.08)	0.035 (0.010)	0.28 (0.01)	1.76 (0.11)	0.73 (0.11)
	**Mid**	2.88 (0.09)	0.050 (0.003)	0.29 (0.01)	2.00 (0.10)	0.57 (0.06)
	**End**	2.87 (0.10)	0.059 (0.005)	0.30 (0.01)	2.39 (0.13)	0.55 (0.05)

Note: Beg = Beginning puff block. Mid = Middle puff block. End = End puff block. BLOD = Below Limit of detection. BLOQ = Below Limit of quantitation.

Table S7. Mean and standard deviation of primary chemical constituent yields per puff in aerosol from eight closed system ends products using whole pod, EWP, and beginning puff block measurements**.**

Product	Measurement	Glycerol	Menthol	Nicotine	Propylene Glycol	Water
		mg/puff	*mg/puff*	*mg/puff*	*mg/puff*	*mg/puff*
JUUL Tobacco	WP	0.79 (0.07)	BLOD	0.061 (0.005)	0.30 (0.03)	0.16 (0.01)
	EWP	0.93 (0.13)	BLOD	0.071 (0.011)	0.36 (0.05)	0.19 (0.02)
	Beg	0.76 (0.16)	BLOD	0.055 (0.013)	0.25 (0.06)	0.20 (0.01)
JUUL Mint/Menthol	WP	0.67 (0.07)	0.009 (0.001)	0.054 (0.006)	0.27 (0.03)	0.14 (0.01)
	EWP	0.76 (0.12)	BLOQ	0.060 (0.009)	0.31 (0.06)	0.18 (0.02)
	Beg	0.72 (0.18)	BLOQ	0.053 (0.010)	0.24 (0.05)	0.20 (0.02)
myBlu Tobacco	WP	2.89 (0.13)	BLOD	0.11 (0.01)	1.99 (0.19)	0.58 (0.01)
	EWP	3.11 (0.11)	BLOD	0.12 (0.004)	2.13 (0.09)	0.86 (0.13)
	Beg	3.12 (0.27)	BLOD	0.12 (0.01)	1.92 (0.12)	0.88 (0.03)
myBlu Mint/Menthol	WP	2.89 (0.04)	0.029 (0.001)	0.10 (0.001)	1.29 (0.01)	0.46 (0.01)
	EWP	2.72 (0.15)	BLOQ	0.093 (0.007)	1.19 (0.10)	0.43 (0.04)
	Beg	3.40 (0.26)	BLOQ	0.11 (0.01)	1.36 (0.12)	0.59 (0.03)
NJOY Ace Tobacco	WP	1.92 (0.17)	BLOD	0.20 (0.02)	1.31 (0.12)	0.61 (0.03)
	EWP	2.49 (0.07)	BLOD	0.24 (0.01)	1.68 (0.07)	0.62 (0.10)
	Beg	2.92 (0.15)	BLOD	0.27 (0.01)	1.72 (0.09)	0.78 (0.07)
NJOY Ace Mint/Menthol	WP	1.85 (0.04)	0.010 (0.0004)	0.19 (0.002)	1.30 (0.03)	0.58 (0.03)
	EWP	1.90 (0.22)	BLOQ	0.19 (0.02)	1.34 (0.21)	0.65 (0.04)
	Beg	2.67 (0.12)	BLOQ	0.25 (0.02)	1.67 (0.12)	0.84 (0.04)
Vuse Alto Tobacco	WP	1.73 (0.20)	BLOD	1.51 (0.88)	0.18 (0.02)	0.45 (0.02)
	EWP	2.19 (0.07)	BLOD	0.22 (0.01)	1.65 (0.09)	0.58 (0.04)
	Beg	2.92 (0.25)	BLOD	0.28 (0.03)	1.89 (0.17)	0.73 (0.06)
Vuse Alto Mint/Menthol	WP	2.40 (0.05)	0.047 (0.001)	0.25 (0.005)	1.74 (0.07)	0.48 (0.02)
	EWP	2.91 (0.05)	0.048 (0.004)	0.29 (0.01)	2.05 (0.07)	0.61 (0.07)
	Beg	2.99 (0.08)	0.035 (0.010)	0.28 (0.01)	1.76 (0.11)	0.73 (0.11)

Note: WP = Whole pod measurement. EWP = Extrapolated whole pod measurement. Beg = Beginning puff block. BLOD = Below Limit of detection. BLOQ = Below Limit of quantitation.

Table S8. Mean and standard deviation of metal chemical constituent measurements per g DML in aerosol from eight closed system ends products during beginning, middle, and end puff blocks.

Product	Puff Block	Arsenic	Beryllium	Cadmium	Chromium	Cobalt	Copper	Iron	Lead	Nickel	Selenium	Silver	Tin	Zinc
		ng/g DML	*ng/g DML*	*ng/g DML*	*ng/g DML*	*ng/g DML*	*ng/g DML*	*ng/g DML*	*ng/g DML*	*ng/g DML*	*ng/g DML*	*ng/g DML*	*ng/g DML*	*ng/g DML*
**JUUL Tobacco**	**Beg**	BLOD	BLOD	BLOD	BLOD	BLOD	BLOD	BLOD	BLOD	BLOQ	BLOQ	BLOD	BLOD	BLOD
	**Mid**	BLOD	BLOD	BLOD	BLOD	BLOD	BLOD	BLOD	BLOD	BLOD	BLOQ	BLOD	BLOD	BLOD
	**End**	BLOD	BLOD	BLOD	BLOQ	BLOD	BLOD	BLOQ	BLOD	BLOD	BLOD	BLOD	BLOD	BLOD
**JUUL Mint/Menthol**	**Beg**	BLOD	BLOD	BLOD	BLOD	BLOD	BLOQ	6349.90 (3113.38)	BLOQ	BLOD	BLOQ	BLOD	BLOD	BLOD
	**Mid**	BLOD	BLOD	BLOD	BLOD	BLOD	BLOD	BLOD	BLOD	BLOD	BLOQ	BLOD	BLOD	BLOD
	**End**	BLOD	BLOD	BLOD	BLOD	BLOD	BLOD	BLOD	BLOD	BLOD	BLOQ	BLOQ	BLOQ	BLOD
**myBlu Tobacco**	**Beg**	BLOQ	BLOD	BLOD	192.12 (150.23)	BLOQ	BLOQ	848.70 (739.05)	37.56 (66.84)	1581.94 (1051.17)	BLOQ	BLOD	BLOQ	BLOD
	**Mid**	BLOD	BLOD	BLOD	BLOQ	BLOQ	BLOD	BLOQ	BLOQ	470.39 (411.72)	BLOQ	BLOQ	BLOQ	BLOD
	**End**	BLOD	BLOD	BLOQ	2224.11 (2474.93)	BLOQ	BLOD	BLOQ	41.72 (44.12)	548.58 (482.53)	BLOQ	BLOD	BLOQ	BLOD
**myBlu Mint/Menthol**	**Beg**	BLOD	BLOD	BLOD	BLOD	BLOD	BLOD	BLOD	BLOD	BLOD	BLOQ	BLOD	BLOD	BLOD
	**Mid**	BLOD	BLOD	BLOD	BLOD	BLOD	BLOD	BLOD	BLOD	BLOD	BLOQ	BLOD	BLOD	BLOD
	**End**	BLOD	BLOD	BLOD	1908.10 (1838.93)	BLOD	BLOD	BLOD	BLOQ	BLOD	BLOD	BLOD	BLOQ	BLOD
**NJOY Ace Tobacco**	**Beg**	BLOD	BLOD	BLOD	BLOQ	BLOQ	667.55 (66.61)	1287.58 (1706.57)	507.26 (173.60)	2144.17 (475.67)	BLOQ	BLOD	BLOQ	4820.78 (900.29)
	**Mid**	BLOD	BLOD	BLOD	BLOQ	BLOD	353.98 (97.46)	BLOQ	716.24 (276.61)	2210.03 (827.35)	BLOQ	BLOD	BLOQ	7026.42 (1637.27)
	**End**	BLOD	BLOD	BLOD	BLOQ	BLOQ	BLOQ	BLOD	825.85 (436.38)	1157.68 (695.52)	BLOQ	BLOD	BLOQ	3890.61 (2386.91)
**NJOY Ace Mint/Menthol**	**Beg**	BLOQ	BLOD	BLOD	BLOQ	BLOD	970.58 (942.60)	BLOD	BLOD	1460.75 (287.50)	88.02 (55.47)	BLOQ	BLOQ	9354.74 (9240.59)
	**Mid**	BLOD	BLOD	BLOD	BLOQ	BLOQ	483.84 (147.34)	BLOQ	BLOQ	1507.27 (872.86)	BLOQ	BLOQ	BLOQ	10565.34 (5781.48)
	**End**	BLOD	BLOD	BLOD	BLOD	BLOQ	BLOD	BLOD	BLOQ	243.05 (120.09)	BLOQ	BLOQ	BLOQ	6103.96 (2624.09)
**Vuse Alto Tobacco**	**Beg**	BLOD	BLOD	BLOD	BLOQ	BLOD	608.21 (803.50)	BLOD	329.91 (633.56)	668.04 (264.56)	BLOQ	BLOQ	BLOQ	4092.94 (8008.09)
	**Mid**	BLOD	BLOD	BLOD	BLOQ	BLOD	597.16 (902.74)	BLOD	259.34 (162.68)	165.72 (125.20)	BLOQ	BLOQ	BLOQ	2303.49 (1487.77)
	**End**	BLOD	BLOD	BLOD	BLOD	BLOD	BLOD	BLOD	64.84 (50.65)	BLOQ	BLOQ	BLOQ	BLOD	BLOD
**Vuse Alto Mint/Menthol**	**Beg**	BLOD	BLOD	BLOD	BLOQ	BLOD	1629.49 (1042.06)	BLOD	1906.85 (1157.98)	1046.17 (426.78)	BLOQ	BLOQ	BLOQ	18592.14 (9738.60)
	**Mid**	BLOD	BLOD	BLOD	BLOQ	BLOQ	930.22 (756.92)	BLOD	2087.88 (1214.05)	1056.68 (485.74)	BLOQ	BLOQ	BLOQ	18263.78 (10588.05)
	**End**	BLOD	BLOD	BLOD	BLOQ	BLOD	342.06 (353.24)	BLOD	1003.83 (564.61)	516.49 (160.61)	BLOQ	BLOQ	BLOQ	8875.72 (4979.19)

Note: DML = Device mass loss. Beg = Beginning puff block. Mid = Middle puff block. End = End puff block. BLOD = Below Limit of detection. BLOQ = Below Limit of quantitation.

Table S9. Mean and standard deviation of metal chemical constituent yields per g DML in aerosol from eight closed system ends products using whole pod, EWP, and beginning puff block measurements.

Product	Measurement	Arsenic	Beryllium	Cadmium	Chromium	Cobalt	Copper	Iron	Lead	Nickel	Selenium	Silver	Tin	Zinc
		ng/g DML	*ng/g DML*	*ng/g DML*	*ng/g DML*	*ng/g DML*	*ng/g DML*	*ng/g DML*	*ng/g DML*	*ng/g DML*	*ng/g DML*	*ng/g DML*	*ng/g DML*	*ng/g DML*
**JUUL Tobacco**	**WP**	BLOD	BLOD	BLOD	BLOD	BLOD	BLOD	BLOD	BLOD	BLOD	BLOQ	BLOD	BLOD	BLOD
	**EWP**	BLOD	BLOD	BLOD	BLOD	BLOD	BLOD	BLOD	BLOD	BLOD	BLOD	BLOD	BLOD	BLOD
	**Beg**	BLOD	BLOD	BLOD	BLOD	BLOD	BLOD	BLOD	BLOD	BLOQ	BLOQ	BLOD	BLOD	BLOD
**JUUL Mint/Menthol**	**WP**	BLOD	BLOD	BLOD	BLOD	BLOD	BLOD	BLOD	BLOD	BLOD	BLOQ	BLOD	BLOD	BLOD
	**EWP**	BLOD	BLOD	BLOD	BLOD	BLOD	BLOD	BLOQ	BLOD	BLOD	BLOQ	BLOD	BLOD	BLOD
	**Beg**	BLOD	BLOD	BLOD	BLOD	BLOD	BLOQ	6350 (3100)	BLOQ	BLOD	BLOQ	BLOD	BLOD	BLOD
**myBlu Tobacco**	**WP**	BLOQ	BLOD	BLOD	239 (180)	BLOQ	BLOD	406 (140)	BLOQ	180 (65)	BLOQ	BLOD	BLOD	BLOD
	**EWP**	BLOD	BLOD	BLOD	829 (830)	BLOQ	BLOD	BLOQ	BLOQ	867 (560)	BLOQ	BLOD	BLOQ	BLOD
	**Beg**	BLOQ	BLOD	BLOD	192 (150)	BLOQ	BLOQ	849 (740)	37.6 (66)	1580 (1100)	BLOQ	BLOD	BLOQ	BLOD
**myBlu Mint/Menthol**	**WP**	BLOQ	BLOD	BLOD	48.9 (55.8)	BLOD	BLOQ	BLOQ	29.1 (48.5)	BLOQ	15.0 (11.9)	BLOD	BLOQ	BLOQ
	**EWP**	BLOD	BLOD	BLOD	BLOQ	BLOD	BLOD	BLOD	BLOD	BLOD	BLOQ	BLOD	BLOD	BLOD
	**Beg**	BLOD	BLOD	BLOD	BLOD	BLOD	BLOD	BLOD	BLOD	BLOD	BLOQ	BLOD	BLOD	BLOD
**NJOY Ace Tobacco**	**WP**	BLOD	BLOD	BLOD	115 (20)	BLOQ	377 (150)	280 (37)	540 (190)	2390 (610)	BLOQ	BLOD	64.6 (30)	5050 (1100)
	**EWP**	BLOD	BLOD	BLOD	BLOQ	BLOQ	411 (64)	BLOQ	683 (200)	1840 (540)	BLOQ	BLOD	BLOQ	5250 (1000)
	**Beg**	BLOD	BLOD	BLOD	BLOQ	BLOQ	668 (67)	1290 (1700)	507 (170)	2140 (480)	BLOQ	BLOD	BLOQ	4820 (900)
**NJOY Ace Mint/Menthol**	**WP**	BLOD	BLOD	BLOD	64.4 (38)	BLOQ	587 (400)	175 (83)	BLOQ	809 (380)	BLOQ	BLOD	58.6 (21)	11600 (6000)
	**EWP**	BLOD	BLOD	BLOD	BLOQ	BLOD	666 (280)	BLOD	BLOQ	973 (120)	BLOQ	BLOQ	BLOQ	10000 (4400)
	**Beg**	BLOQ	BLOD	BLOD	BLOQ	BLOD	971 (940)	BLOD	BLOD	1460 (290)	88.0 (55)	BLOQ	BLOQ	9350 (9200)
**Vuse Alto Tobacco**	**WP**	BLOD	BLOD	BLOD	BLOQ	BLOD	459 (390)	BLOD	353 (360)	363 (220)	BLOQ	BLOD	BLOQ	4060 (4100)
	**EWP**	BLOD	BLOD	BLOD	BLOQ	BLOD	433 (360)	BLOD	218 (250)	287 (85)	BLOQ	BLOQ	BLOD	2390 (3100)
	**Beg**	BLOD	BLOD	BLOD	BLOQ	BLOD	608 (800)	BLOD	330 (634)	668 (260)	BLOQ	BLOQ	BLOQ	4090 (8010)
**Vuse Alto Mint/Menthol**	**WP**	BLOQ	BLOD	BLOD	71.6 (5.2)	BLOD	232 (120)	152 (22)	30.6 (67)	836 (100)	BLOQ	BLOD	BLOQ	1110 (390)
	**EWP**	BLOD	BLOD	BLOD	BLOQ	BLOD	967 (510)	BLOD	1670 (940)	873 (270)	BLOQ	BLOQ	BLOQ	15200 (8000)
	**Beg**	BLOD	BLOD	BLOD	BLOQ	BLOD	1630 (1000)	BLOD	1910 (1200)	1050 (430)	BLOQ	BLOQ	BLOQ	18600 (10000)

Note: DML = Device mass loss. WP = Whole pod measurement. EWP = Extrapolated whole pod measurement. Beg = Beginning puff block. BLOD = Below Limit of detection. BLOQ = Below Limit of quantitation.

Table S10. Mean and standard deviation of metal chemical constituent measurements per puff in aerosol from eight closed system ends products during beginning, middle, and end puff blocks.

Product	Puff Block	Arsenic	Beryllium	Cadmium	Chromium	Cobalt	Copper	Iron	Lead	Nickel	Selenium	Silver	Tin	Zinc
		ng/puff	*ng/puff*	*ng/puff*	*ng/puff*	*ng/puff*	*ng/puff*	*ng/puff*	*ng/puff*	*ng/puff*	*ng/puff*	*ng/puff*	*ng/puff*	*ng/puff*
**JUUL Tobacco**	**Beg**	BLOD	BLOD	BLOD	BLOD	BLOD	BLOD	BLOD	BLOD	BLOQ	BLOQ	BLOD	BLOD	BLOD
	**Mid**	BLOD	BLOD	BLOD	BLOD	BLOD	BLOD	BLOD	BLOD	BLOD	BLOQ	BLOD	BLOD	BLOD
	**End**	BLOD	BLOD	BLOD	BLOQ	BLOD	BLOD	BLOQ	BLOD	BLOD	BLOD	BLOD	BLOD	BLOD
**JUUL Mint/Menthol**	**Beg**	BLOD	BLOD	BLOD	BLOD	BLOD	BLOQ	5.44 (2.73)	BLOQ	BLOD	BLOQ	BLOD	BLOD	BLOD
	**Mid**	BLOD	BLOD	BLOD	BLOD	BLOD	BLOD	BLOD	BLOD	BLOD	BLOQ	BLOD	BLOD	BLOD
	**End**	BLOD	BLOD	BLOD	BLOD	BLOD	BLOD	BLOD	BLOD	BLOD	BLOQ	BLOQ	BLOQ	BLOD
**myBlu Tobacco**	**Beg**	BLOQ	BLOD	BLOD	1.21 (0.90)	BLOQ	BLOQ	5.34 (4.44)	0.23 (0.40)	9.82 (6.35)	BLOQ	BLOD	BLOQ	BLOD
	**Mid**	BLOD	BLOD	BLOD	BLOQ	BLOQ	BLOD	BLOQ	BLOQ	3.46 (3.19)	BLOQ	BLOQ	BLOQ	BLOD
	**End**	BLOD	BLOD	BLOQ	7.02 (8.04)	BLOQ	BLOD	BLOQ	0.22 (0.33)	3.52 (3.54)	BLOQ	BLOD	BLOQ	BLOD
**myBlu Mint/Menthol**	**Beg**	BLOD	BLOD	BLOD	BLOD	BLOD	BLOD	BLOD	BLOD	BLOD	BLOQ	BLOD	BLOD	BLOD
	**Mid**	BLOD	BLOD	BLOD	BLOD	BLOD	BLOD	BLOD	BLOD	BLOD	BLOQ	BLOD	BLOD	BLOD
	**End**	BLOD	BLOD	BLOD	2.18 (2.95)	BLOD	BLOD	BLOD	BLOQ	BLOD	BLOD	BLOD	BLOQ	BLOD
**NJOY Ace Tobacco**	**Beg**	BLOD	BLOD	BLOD	BLOQ	BLOQ	3.78 (0.45)	7.08 (9.12)	2.86 (0.93)	12.11 (2.66)	BLOQ	BLOD	BLOQ	27.18 (4.62)
	**Mid**	BLOD	BLOD	BLOD	BLOQ	BLOD	1.93 (0.56)	BLOQ	3.91 (1.59)	12.13 (4.85)	BLOQ	BLOD	BLOQ	38.20 (9.01)
	**End**	BLOD	BLOD	BLOD	BLOQ	BLOQ	BLOQ	BLOD	2.94 (1.63)	4.23 (3.03)	BLOQ	BLOD	BLOQ	14.42 (10.79)
**NJOY Ace Mint/Menthol**	**Beg**	BLOQ	BLOD	BLOD	BLOQ	BLOD	5.20 (5.08)	BLOD	BLOD	7.95 (1.75)	0.47 (0.26)	BLOQ	BLOQ	49.98 (49.53)
	**Mid**	BLOD	BLOD	BLOD	BLOQ	BLOQ	2.30 (0.33)	BLOD	BLOQ	7.87 (5.45)	BLOQ	BLOQ	BLOQ	48.66 (20.40)
	**End**	BLOD	BLOD	BLOD	BLOD	BLOQ	BLOD	BLOD	BLOQ	0.47 (0.43)	BLOQ	BLOQ	BLOQ	10.81 (8.59)
**Vuse Alto Tobacco**	**Beg**	BLOD	BLOD	BLOD	BLOQ	BLOD	2.65 (3.30)	BLOD	1.40 (2.64)	3.04 (0.96)	BLOQ	BLOQ	BLOQ	17.31 (33.42)
	**Mid**	BLOD	BLOD	BLOD	BLOQ	BLOD	3.06 (5.05)	BLOD	1.18 (0.83)	0.79 (0.73)	BLOQ	BLOQ	BLOQ	10.37 (7.07)
	**End**	BLOD	BLOD	BLOD	BLOD	BLOD	BLOD	BLOD	0.25 (0.17)	BLOQ	BLOQ	BLOQ	BLOD	BLOD
**Vuse Alto Mint/Menthol**	**Beg**	BLOD	BLOD	BLOD	BLOQ	BLOD	8.67 (5.61)	BLOD	10.19 (6.38)	5.44 (2.45)	BLOQ	BLOQ	BLOQ	99.38 (56.07)
	**Mid**	BLOD	BLOD	BLOD	BLOQ	BLOQ	5.43 (4.70)	BLOD	12.09 (7.53)	6.08 (2.75)	BLOQ	BLOQ	BLOQ	105.46 (65.08)
	**End**	BLOD	BLOD	BLOD	BLOQ	BLOD	1.90 (2.02)	BLOD	5.54 (3.30)	2.92 (1.11)	BLOQ	BLOQ	BLOQ	48.76 (28.87)

Note: Beg = Beginning puff block. Mid = Middle puff block. End = End puff block. BLOD = Below Limit of detection. BLOQ = Below Limit of quantitation.

Table S11. Mean and standard deviation of metal chemical constituent yields per puff in aerosol from eight closed system ends products using whole pod, EWP, and beginning puff block measurements**.**

Product	Measurement	Arsenic	Beryllium	Cadmium	Chromium	Cobalt	Copper	Iron	Lead	Nickel	Selenium	Silver	Tin	Zinc
		ng/puff	*ng/puff*	*ng/puff*	*ng/puff*	*ng/puff*	*ng/puff*	*ng/puff*	*ng/puff*	*ng/puff*	*ng/puff*	*ng/puff*	*ng/puff*	*ng/puff*
**JUUL Tobacco**	**WP**	BLOD	BLOD	BLOD	BLOD	BLOD	BLOD	BLOD	BLOD	BLOD	BLOQ	BLOD	BLOD	BLOD
	**EWP**	BLOD	BLOD	BLOD	BLOD	BLOD	BLOD	BLOD	BLOD	BLOD	BLOQ	BLOD	BLOD	BLOD
	**Beg**	BLOD	BLOD	BLOD	BLOD	BLOD	BLOD	BLOD	BLOD	BLOQ	BLOQ	BLOD	BLOD	BLOD
**JUUL Mint/Menthol**	**WP**	BLOD	BLOD	BLOD	BLOD	BLOD	BLOD	BLOD	BLOD	BLOD	BLOQ	BLOD	BLOD	BLOD
	**EWP**	BLOD	BLOD	BLOD	BLOD	BLOD	BLOD	BLOQ	BLOD	BLOD	BLOQ	BLOD	BLOD	BLOD
	**Beg**	BLOD	BLOD	BLOD	BLOD	BLOD	BLOQ	5.44 (2.73)	BLOQ	BLOD	BLOQ	BLOD	BLOD	BLOD
**myBlu Tobacco**	**WP**	BLOQ	BLOD	BLOD	1.47 (1.14)	BLOQ	BLOD	2.46 (0.87)	BLOQ	1.10 (0.42)	BLOQ	BLOD	BLOD	BLOD
	**EWP**	BLOD	BLOD	BLOD	2.91 (2.47)	BLOQ	BLOD	BLOQ	BLOQ	5.60 (3.87)	BLOQ	BLOD	BLOQ	BLOD
	**Beg**	BLOQ	BLOD	BLOD	1.21 (0.90)	BLOQ	BLOQ	5.34 (4.44)	0.23 (0.40)	9.82 (6.35)	BLOQ	BLOD	BLOQ	BLOD
**myBlu Mint/Menthol**	**WP**	BLOQ	BLOD	BLOD	0.24 (0.28)	BLOD	BLOQ	BLOQ	0.15 (0.24)	BLOQ	0.075 (0.060)	BLOD	BLOQ	BLOQ
	**EWP**	BLOD	BLOD	BLOD	BLOQ	BLOD	BLOD	BLOD	BLOD	BLOD	BLOQ	BLOD	BLOD	BLOD
	**Beg**	BLOD	BLOD	BLOD	BLOD	BLOD	BLOD	BLOD	BLOD	BLOD	BLOQ	BLOD	BLOD	BLOD
**NJOY Ace Tobacco**	**WP**	BLOD	BLOD	BLOD	0.59 (0.10)	BLOQ	1.93 (0.80)	1.43 (0.21)	2.77 (1.00)	12.13 (2.71)	BLOQ	BLOD	0.33 (0.15)	25.87 (5.93)
	**EWP**	BLOD	BLOD	BLOD	BLOQ	BLOQ	2.14 (0.25)	BLOQ	3.23 (1.16)	9.49 (2.99)	BLOQ	BLOD	BLOQ	26.60 (5.74)
	**Beg**	BLOD	BLOD	BLOD	BLOQ	BLOQ	3.78 (0.45)	7.08 (9.12)	2.86 (0.93)	12.11 (2.66)	BLOQ	BLOD	BLOQ	27.18 (4.62)
**NJOY Ace Mint/Menthol**	**WP**	BLOD	BLOD	BLOD	0.29 (0.19)	BLOQ	2.45 (1.52)	0.76 (0.41)	BLOQ	3.57 (1.92)	BLOQ	BLOD	0.25 (0.07)	48.55 (21.93)
	**EWP**	BLOD	BLOD	BLOD	BLOQ	BLOD	3.00 (1.72)	BLOD	BLOQ	4.78 (0.77)	BLOQ	BLOQ	BLOQ	42.87 (21.56)
	**Beg**	BLOQ	BLOD	BLOD	BLOQ	BLOD	5.20 (5.08)	BLOD	BLOD	7.95 (1.75)	0.47 (0.26)	BLOQ	BLOQ	49.98 (49.53)
**Vuse Alto Tobacco**	**WP**	BLOD	BLOD	BLOD	BLOQ	BLOD	1.81 (1.39)	BLOD	1.38 (1.35)	1.51 (0.88)	BLOQ	BLOD	BLOQ	15.91 (15.57)
	**EWP**	BLOD	BLOD	BLOD	BLOQ	BLOD	2.04 (1.85)	BLOD	0.94 (1.01)	1.31 (0.33)	BLOQ	BLOQ	BLOD	10.19 (12.60)
	**Beg**	BLOD	BLOD	BLOD	BLOQ	BLOD	2.65 (3.30)	BLOD	1.40 (2.64)	3.04 (0.96)	BLOQ	BLOQ	BLOQ	17.31 (33.42)
**Vuse Alto Mint/Menthol**	**WP**	BLOQ	BLOD	BLOD	0.45 (0.04)	BLOD	1.44 (0.70)	0.95 (0.13)	0.20 (0.43)	5.23 (0.61)	BLOQ	BLOD	BLOQ	6.90 (2.29)
	**EWP**	BLOD	BLOD	BLOD	BLOQ	BLOD	5.34 (2.96)	BLOD	9.27 (5.45)	4.81 (1.35)	BLOQ	BLOQ	BLOQ	84.53 (46.92)
	**Beg**	BLOD	BLOD	BLOD	BLOQ	BLOD	8.67 (5.61)	BLOD	10.19 (6.38)	5.44 (2.45)	BLOQ	BLOQ	BLOQ	99.38 (56.07)

Note: WP = Whole pod measurement. EWP = Extrapolated whole pod measurement. FP = First fifty puff measurement. BLOD = Below Limit of detection. BLOQ = Below Limit of quantitation.

Table S12. Mean and standard deviation of carbonyl chemical constituent measurements per g DML in aerosol from eight closed system ends products during beginning, middle, and end puff blocks.

Product	Puff Block	Acetaldehyde	Acetyl Propionyl	Acrolein	n-Butyraldehyde	Crotonaldehyde	Diacetyl	Formaldehyde
		μg/g DML	*μg/g DML*	*μg/g DML*	*μg/g DML*	*μg/g DML*	*μg/g DML*	*μg/g DML*
**JUUL Tobacco**	**Beg**	BLOQ	BLOD	BLOD	BLOD	BLOD	BLOD	BLOQ
	**Mid**	BLOQ	BLOD	BLOQ	BLOD	BLOD	BLOD	37.74 (18.26)
	**End**	BLOQ	BLOD	BLOQ	BLOD	BLOD	BLOD	34.35 (13.67)
**JUUL Mint/Menthol**	**Beg**	BLOQ	BLOD	BLOD	BLOD	BLOD	BLOD	37.26 (8.07)
	**Mid**	BLOQ	BLOD	BLOQ	BLOD	BLOD	BLOD	39.62 (7.59)
	**End**	BLOQ	BLOD	BLOQ	BLOD	BLOD	BLOD	41.22 (4.47)
**myBlu Tobacco**	**Beg**	238.39 (291.51)	BLOQ	99.58 (136.12)	BLOQ	BLOQ	8.22 (5.79)	383.60 (616.54)
	**Mid**	111.94 (30.09)	BLOQ	61.45 (25.78)	BLOQ	BLOD	8.52 (3.66)	70.70 (49.87)
	**End**	132.97 (55.64)	BLOQ	78.48 (34.51)	BLOQ	BLOD	13.33 (6.38)	61.30 (41.76)
**myBlu Mint/Menthol**	**Beg**	24.15 (14.48)	BLOD	23.06 (10.37)	BLOD	BLOD	BLOQ	68.58 (53.07)
	**Mid**	16.95 (6.13)	BLOD	20.91 (6.16)	BLOD	BLOD	BLOQ	25.94 (13.74)
	**End**	12472.97 (16987.36	BLOQ	7470.30 (9708.70)	48.84 (55.83)	49.77 (41.89)	97.01 (150.06)	16126.61 (19138.05)
**NJOY Ace Tobacco**	**Beg**	64.06 (5.21)	BLOQ	14.97 (3.56)	BLOD	BLOD	BLOQ	12.10 (1.07)
	**Mid**	73.96 (8.60)	BLOQ	34.28 (13.03)	BLOD	BLOD	8.16 (4.28)	17.32 (3.63)
	**End**	137.38 (87.64)	6.75 (5.09)	90.93 (66.35)	BLOQ	BLOD	24.16 (19.10)	62.89 (60.31)
**NJOY Ace Mint/Menthol**	**Beg**	52.68 (29.66)	BLOQ	13.61 (10.22)	BLOD	BLOD	4.58 (3.12)	35.43 (22.13)
	**Mid**	72.11 (51.40)	BLOQ	42.17 (37.78)	BLOQ	BLOQ	14.69 (14.23)	60.10 (45.39)
	**End**	1441.58 (2108.46)	19.48 (13.61)	557.11 (797.17)	BLOQ	BLOQ	78.61 (69.13)	2077.73 (3236.45)
**Vuse Alto Tobacco**	**Beg**	43.49 (20.75)	BLOQ	12.04 (8.55)	BLOQ	BLOD	4.20 (2.76)	45.90 (22.20)
	**Mid**	53.50 (31.92)	BLOQ	28.36 (19.66)	BLOQ	BLOQ	8.51 (8.12)	38.87 (17.48)
	**End**	327.26 (459.94)	7.61 (5.20)	108.37 (131.32)	BLOQ	BLOQ	29.69 (22.39)	601.35 (1066.85)
**Vuse Alto Mint/Menthol**	**Beg**	21.38 (1.73)	BLOQ	5.30 (1.16)	BLOD	BLOD	BLOQ	8.65 (1.34)
	**Mid**	13.09 (1.22)	BLOQ	5.87 (1.03)	BLOD	BLOD	BLOQ	8.50 (1.08)
	**End**	13.87 (1.17)	BLOQ	4.49 (2.54)	BLOD	BLOD	BLOQ	8.59 (0.97)

Note: DML = Device mass loss. Beg = Beginning puff block. Mid = Middle puff block. End = End puff block. BLOD = Below Limit of detection. BLOQ = Below Limit of quantitation.

Table S13. Mean and standard deviation of carbonyl chemical constituent yields per g DML in aerosol from eight closed system ends products using whole pod, EWP, and beginning puff block measurements.

Product	Measurement	Acetaldehyde	Acetyl Propionyl	Acrolein	n-Butyraldehyde	Crotonaldehyde	Diacetyl	Formaldehyde
		μg/g DML	*μg/g DML*	*μg/g DML*	*μg/g DML*	*μg/g DML*	*μg/g DML*	*μg/g DML*
**JUUL Tobacco**	**WP**	13.4 (6.2)	BLOD	7.24 (1.9)	BLOD	BLOD	BLOQ	65.7 (28)
	**EWP**	BLOQ	BLOD	BLOQ	BLOD	BLOD	BLOD	30.8 (8.8)
	**Beg**	BLOQ	BLOD	BLOD	BLOD	BLOD	BLOD	BLOQ
**JUUL Mint/Menthol**	**WP**	11.4 (1.3)	BLOD	5.83 (0.51)	BLOQ	BLOD	BLOQ	45.8 (12)
	**EWP**	BLOQ	BLOD	BLOQ	BLOD	BLOD	BLOD	39.4 (4.8)
	**Beg**	BLOQ	BLOD	BLOD	BLOD	BLOD	BLOD	37.3 (8.1)
**myBlu Tobacco**	**WP**	353 (160)	2.50 (0.44)	103 (42)	1.49 (1.0)	1.23 (0.66)	12.5 (2.5)	795 (430)
	**EWP**	161 (110)	BLOQ	79.8 (57.6)	BLOQ	BLOD	10.0 (4.6)	172 (230)
	**Beg**	238 (290)	BLOQ	99.6 (140)	BLOQ	BLOQ	8.22 (5.8)	384 (620)
**myBlu Mint/Menthol**	**WP**	398 (61)	BLOQ	247 (26)	2.72 (0.52)	2.99 (0.37)	3.87 (0.55)	530 (98)
	**EWP**	4170 (5700)	BLOQ	2500 (3200)	BLOQ	BLOQ	33.7 (50)	5410 (6400)
	**Beg**	24.2 (15)	BLOD	23.1 (10)	BLOD	BLOD	BLOQ	68.6 (53)
**NJOY Ace Tobacco**	**WP**	94.5 (29)	2.82 (1.0)	42.1 (19)	BLOQ	BLOQ	10.4 (3.7)	57.3 (48)
	**EWP**	91.8 (32.0)	BLOQ	46.7 (26)	BLOQ	BLOD	11.6 (7.5)	30.8 (21)
	**Beg**	64.1 (5.2)	BLOQ	15.0 (3.6)	BLOD	BLOD	BLOQ	12.1 (1.1)
**NJOY Ace Mint/Menthol**	**WP**	92.0 (23)	4.63 (2.1)	46.9 (20)	BLOQ	BLOQ	18.7 (7.4)	65.6 (16)
	**EWP**	538 (680)	9.13 (3.6)	213 (250)	BLOQ	BLOQ	35.9 (18)	740 (1100)
	**Beg**	52.7 (30)	BLOQ	13.6 (10)	BLOD	BLOD	4.58 (3.1)	35.4 (22)
**Vuse Alto Tobacco**	**WP**	63.9 (50)	4.72 (3.9)	39.6 (31)	1.26 (0.50)	BLOQ	14.7 (14)	35.8 (35)
	**EWP**	141 (150)	BLOQ	49.6 (44)	BLOQ	BLOQ	14.1 (11)	229 (360)
	**Beg**	43.5 (21)	BLOQ	12.0 (8.6)	BLOQ	BLOD	4.20 (2.8)	45.9 (22)
**Vuse Alto Mint/Menthol**	**WP**	17.5 (3.5)	1.06 (0.32)	5.85 (2.0)	BLOQ	BLOD	1.07 (0.64)	10.0 (3.1)
	**EWP**	16.0 (1.3)	BLOQ	5.22 (1.1)	BLOD	BLOD	BLOQ	8.58 (0.95)
	**Beg**	21.4 (1.7)	BLOQ	5.30 (1.2)	BLOD	BLOD	BLOQ	8.65 (1.3)

Note: DML = Device mass loss. WP = Whole pod measurement. EWP = Extrapolated whole pod measurement. Beg = Beginning puff block. BLOD = Below Limit of detection. BLOQ = Below Limit of quantitation.

Table S14. Mean and standard deviation of carbonyl chemical constituent measurements per puff in aerosol from eight closed system ends products during beginning, middle, and end puff blocks.

**Product**	**Puff Block**	**Acetaldehyde**	**Acetyl Propionyl**	**Acrolein**	**n-Butyraldehyde**	**Crotonaldehyde**	**Diacetyl**	**Formaldehyde**
		μg/puff	*μg/puff*	*μg/puff*	*μg/puff*	*μg/puff*	*μg/puff*	*μg/puff*
**JUUL Tobacco**	**Beg**	BLOQ	BLOD	BLOD	BLOD	BLOD	BLOD	BLOQ
	**Mid**	BLOQ	BLOD	BLOQ	BLOD	BLOD	BLOD	0.052 (0.028)
	**End**	BLOQ	BLOD	BLOQ	BLOD	BLOD	BLOD	0.059 (0.033)
**JUUL Mint/Menthol**	**Beg**	BLOQ	BLOD	BLOD	BLOD	BLOD	BLOD	0.032 (0.005)
	**Mid**	BLOQ	BLOD	BLOQ	BLOD	BLOD	BLOD	0.062 (0.010)
	**End**	BLOQ	BLOD	BLOQ	BLOD	BLOD	BLOD	0.048 (0.017)
**myBlu Tobacco**	**Beg**	1.47 (1.94)	BLOQ	0.62 (0.90)	BLOQ	BLOQ	0.049 (0.040)	2.42 (4.07)
	**Mid**	0.69 (0.23)	BLOQ	0.38 (0.18)	BLOQ	BLOD	0.053 (0.027)	0.43 (0.31)
	**End**	0.77 (0.37)	BLOQ	0.45 (0.22)	BLOQ	BLOD	0.078 (0.041)	0.35 (0.26)
**myBlu Mint/Menthol**	**Beg**	0.14 (0.08)	BLOD	0.14 (0.06)	BLOD	BLOD	BLOQ	0.40 (0.29)
	**Mid**	0.10 (0.04)	BLOD	0.13 (0.04)	BLOD	BLOD	BLOQ	0.16 (0.08)
	**End**	9.25 (5.71)	BLOQ	5.89 (3.61)	0.044 (0.033)	0.058 (0.042)	0.064 (0.032)	13.98 (9.12)
**NJOY Ace Tobacco**	**Beg**	0.33 (0.03)	BLOQ	0.078 (0.020)	BLOD	BLOD	BLOQ	0.063 (0.007)
	**Mid**	0.37 (0.03)	BLOQ	0.17 (0.06)	BLOD	BLOD	0.040 (0.019)	0.086 (0.014)
	**End**	0.50 (0.19)	0.024 (0.013)	0.32 (0.16)	BLOQ	BLOD	0.085 (0.048)	0.22 (0.15)
**NJOY Ace Mint/Menthol**	**Beg**	0.28 (0.15)	BLOQ	0.071 (0.052)	BLOD	BLOD	0.024 (0.016)	0.19 (0.11)
	**Mid**	0.31 (0.17)	BLOQ	0.18 (0.14)	BLOQ	BLOQ	0.060 (0.053)	0.27 (0.22)
	**End**	2.08 (2.50)	0.037 (0.009)	0.82 (0.94)	BLOQ	BLOQ	0.14 (0.06)	2.88 (3.97)
**Vuse Alto Tobacco**	**Beg**	0.24 (0.11)	BLOQ	0.067 (0.046)	BLOQ	BLOD	0.023 (0.015)	0.26 (0.12)
	**Mid**	0.23 (0.10)	BLOQ	0.12 (0.06)	BLOQ	BLOQ	0.035 (0.027)	0.17 (0.06)
	**End**	1.03 (1.36)	0.025 (0.013)	0.35 (0.39)	BLOQ	BLOQ	0.10 (0.06)	1.87 (3.20)
**Vuse Alto Mint/Menthol**	**Beg**	0.12 (0.01)	BLOQ	0.029 (0.006)	BLOD	BLOD	BLOQ	0.048 (0.006)
	**Mid**	0.079 (0.008)	BLOQ	0.036 (0.007)	BLOD	BLOD	BLOQ	0.052 (0.008)
	**End**	0.082 (0.006)	BLOQ	0.026 (0.014)	BLOD	BLOD	BLOQ	0.051 (0.006)

Note: Beg = Beginning puff block. Mid = Middle puff block. End = End puff block. BLOD = Below Limit of detection. BLOQ = Below Limit of quantitation.

Table S15. Mean and standard deviation of metal chemical constituent yields per puff in aerosol from eight closed system ends products using whole pod, EWP, and beginning puff block measurements.

Product	Measurement	Acetaldehyde	Acetyl Propionyl	Acrolein	n-Butyraldehyde	Crotonaldehyde	Diacetyl	Formaldehyde
		μg/puff	*μg/puff*	*μg/puff*	*μg/puff*	*μg/puff*	*μg/puff*	*μg/puff*
**JUUL Tobacco**	**WP**	0.017 (0.008)	BLOD	0.009 (0.003)	BLOD	BLOD	BLOQ	0.084 (0.042)
	**EWP**	BLOQ	BLOD	BLOQ	BLOD	BLOD	BLOD	0.043 (0.015)
	**Beg**	BLOQ	BLOD	BLOD	BLOD	BLOD	BLOD	BLOQ
**JUUL Mint/Menthol**	**WP**	0.013 (0.003)	BLOD	0.007 (0.001)	BLOQ	BLOD	BLOQ	0.054 (0.019)
	**EWP**	BLOQ	BLOD	BLOQ	BLOD	BLOD	BLOD	0.047 (0.005)
	**Beg**	BLOQ	BLOD	BLOD	BLOD	BLOD	BLOD	0.032 (0.005)
**myBlu Tobacco**	**WP**	2.08 (0.97)	0.015 (0.002)	0.61 (0.26)	0.009 (0.007)	0.007 (0.004)	0.074 (0.013)	4.69 (2.60)
	**EWP**	0.98 (0.73)	BLOQ	0.48 (0.37)	BLOQ	BLOD	0.060 (0.030)	1.07 (1.46)
	**Beg**	1.47 (1.94)	BLOQ	0.62 (0.90)	BLOQ	BLOQ	0.049 (0.040)	2.42 (4.07)
**myBlu Mint/Menthol**	**WP**	2.05 (0.32)	BLOQ	1.27 (0.14)	0.014 (0.003)	0.015 (0.002)	0.020 (0.003)	2.72 (0.51)
	**EWP**	3.16 (1.92)	BLOQ	2.05 (1.22)	BLOQ	BLOQ	0.030 (0.011)	4.85 (3.10)
	**Beg**	0.14 (0.08)	BLOD	0.14 (0.06)	BLOD	BLOD	BLOQ	0.40 (0.29)
**NJOY Ace Tobacco**	**WP**	0.45 (0.22)	0.013 (0.005)	0.20 (0.13)	BLOQ	BLOQ	0.048 (0.020)	0.29 (0.30)
	**EWP**	0.40 (0.08)	BLOQ	0.19 (0.07)	BLOQ	BLOD	0.046 (0.021)	0.12 (0.05)
	**Beg**	0.33 (0.03)	BLOQ	0.078 (0.020)	BLOD	BLOD	BLOQ	0.063 (0.007)
**NJOY Ace Mint/Menthol**	**WP**	0.37 (0.09)	0.018 (0.008)	0.19 (0.08)	BLOQ	BLOQ	0.074 (0.029)	0.26 (0.06)
	**EWP**	0.96 (0.75)	0.024 (0.003)	0.39 (0.26)	BLOQ	BLOQ	0.089 (0.010)	1.18 (1.37)
	**Beg**	0.28 (0.15)	BLOQ	0.071 (0.052)	BLOD	BLOD	0.024 (0.016)	0.19 (0.11)
**Vuse Alto Tobacco**	**WP**	0.23 (0.15)	0.017 (0.012)	0.14 (0.10)	0.005 (0.001)	BLOQ	0.052 (0.044)	0.13 (0.11)
	**EWP**	0.50 (0.46)	BLOQ	0.18 (0.13)	BLOQ	BLOQ	0.052 (0.033)	0.77 (1.07)
	**Beg**	0.24 (0.11)	BLOQ	0.067 (0.046)	BLOQ	BLOD	0.023 (0.015)	0.26 (0.12)
**Vuse Alto Mint/Menthol**	**WP**	0.096 (0.016)	0.006 (0.002)	0.032 (0.011)	BLOQ	BLOD	0.006 (0.003)	0.055 (0.019)
	**EWP**	0.093 (0.007)	BLOQ	0.030 (0.006)	BLOD	BLOD	BLOQ	0.050 (0.006)
	**Beg**	0.12 (0.01)	BLOQ	0.029 (0.006)	BLOD	BLOD	BLOQ	0.048 (0.006)

Note: WP = Whole pod measurement. EWP = Extrapolated whole pod measurement. Beg = Beginning puff block. BLOD = Below Limit of detection. BLOQ = Below Limit of quantitation.

Table S16. Mean and standard deviation of glycidol chemical constituent measurements per g DML in aerosol from eight closed system ends products during beginning, middle, and end puff blocks.

Product	Puff Block	Glycidol
		µg/g DML
JUUL Tobacco	Beg	1.20 (0.94)
	Mid	3.83 (1.53)
	End	4.69 (1.23)
JUUL Mint/Menthol	Beg	2.94 (0.33)
	Mid	7.74 (1.15)
	End	10.08 (6.06)
myBlu Tobacco	Beg	45.44 (45.66)
	Mid	95.10 (77.39)
	End	214.19 (151.38)
myBlu Mint/Menthol	Beg	18.08 (5.72)
	Mid	31.14 (11.23)
	End	744.45 (581.87)
NJOY Ace Tobacco	Beg	20.41 (3.44)
	Mid	26.46 (14.30)
	End	116.45 (50.04)
NJOY Ace Mint/Menthol	Beg	23.07 (13.12)
	Mid	55.42 (41.86)
	End	197.85 (91.54)
Vuse Alto Tobacco	Beg	16.60 (3.87)
	Mid	23.18 (7.57)
	End	73.87 (62.74)
Vuse Alto Mint/Menthol	Beg	12.21 (1.06)
	Mid	10.75 (4.41)
	End	12.16 (3.27)

Note: DML = Device mass loss. Beg = Beginning puff block. Mid = Middle puff block. End = End puff block.

Table S17. Mean and standard deviation of glycidol chemical constituent yields per g DML in aerosol from eight closed system ends products using whole pod, EWP, and beginning puff block measurements.

Product	Measurement	Glycidol
		µg/g DML
JUUL Tobacco	WP	4.95 (0.91)
	EWP	3.24 (0.53)
	Beg	1.20 (0.94)
JUUL Mint/Menthol	WP	5.03 (0.89)
	EWP	7.15 (2.1)
	Beg	2.94 (0.33)
myBlu Tobacco	WP	31.6 (20) [a]
	EWP	118 (83)
	Beg	45.4 (46)
myBlu Mint/Menthol	WP	18.2 (1.7) [a]
	EWP	267 (190)
	Beg	18.1 (5.7)
NJOY Ace Tobacco	WP	16.9 (4.7) [a]
	EWP	54.4 (22)
	Beg	20.4 (3.4)
NJOY Ace Mint/Menthol	WP	9.77 (2.2) [a]
	EWP	104 (42)
	Beg	23.1 (13)
Vuse Alto Tobacco	WP	89.7 (28) [a]
	EWP	37.9 (21)
	Beg	16.6 (3.9)
Vuse Alto Mint/Menthol	WP	45.7 (46) [a]
	EWP	11.7 (2.8)
	Beg	12.2 (1.1)

Note: DML = Device mass loss. WP = Whole pod measurement. EWP = Extrapolated whole pod measurement. Beg = Beginning puff block. [a] WP results use different EOL from EWP, see Table S2

Table S18. Mean and standard deviation of glycidol chemical constituent measurements per puff in aerosol from eight closed system ends products during beginning, middle, and end puff blocks.

Product	Puff Block	Glycidol
		μg/puff
JUUL Tobacco	Beg	0.001 (0.001)
	Mid	0.005 (0.002)
	End	0.007 (0.003)
JUUL Mint/Menthol	Beg	0.003 (0.001)
	Mid	0.011 (0.001)
	End	0.008 (0.004)
myBlu Tobacco	Beg	0.22 (0.19)
	Mid	0.56 (0.42)
	End	0.81 (0.49)
myBlu Mint/Menthol	Beg	0.10 (0.03)
	Mid	0.20 (0.05)
	End	0.40 (0.09)
NJOY Ace Tobacco	Beg	0.11 (0.02)
	Mid	0.13 (0.07)
	End	0.37 (0.10)
NJOY Ace Mint/Menthol	Beg	0.11 (0.06)
	Mid	0.20 (0.11)
	End	1.11 (0.47)
Vuse Alto Tobacco	Beg	0.090 (0.019)
	Mid	0.11 (0.04)
	End	0.25 (0.18)
Vuse Alto Mint/Menthol	Beg	0.068 (0.007)
	Mid	0.060 (0.024)
	End	0.066 (0.018)

Note: Beg = Beginning puff block. Mid = Middle puff block. End = End puff block.

Table S19. Mean and standard deviation of glycidol chemical constituent yields per puff in aerosol from eight closed system ends products using whole pod, EWP, and beginning puff block measurements.

Product	Measurement	Glycidol
		μg/puff
**JUUL Tobacco**	**WP**	0.006 (0.002)
	**EWP**	0.004 (0.001)
	**Beg**	0.001 (0.001)
**JUUL Mint/Menthol**	**WP**	0.006 (0.001)
	**EWP**	0.007 (0.001)
	**Beg**	0.003 (0.001)
**myBlu Tobacco**	**WP**	0.16 (0.10)[a]
	**EWP**	0.53 (0.36)
	**Beg**	0.22 (0.19)
**myBlu Mint/Menthol**	**WP**	0.10 (0.01)[a]
	**EWP**	0.24 (0.03)
	**Beg**	0.10 (0.03)
**NJOY Ace Tobacco**	**WP**	0.10 (0.03)[a]
	**EWP**	0.20 (0.06)
	**Beg**	0.11 (0.02)
**NJOY Ace Mint/Menthol**	**WP**	0.061 (0.015)[a]
	**EWP**	0.31 (0.06)
	**Beg**	0.11 (0.06)
**Vuse Alto Tobacco**	**WP**	0.38 (0.08)[a]
	**EWP**	0.15 (0.06)
	**Beg**	0.090 (0.019)
**Vuse Alto Mint/Menthol**	**WP**	0.18 (0.13)[a]
	**EWP**	0.064 (0.016)
	**Beg**	0.068 (0.007)

Note: WP = Whole pod measurement. EWP = Extrapolated whole pod measurement. Beg = Beginning puff block. [a] WP results use different EOL from EWP, see Table S2
